# Heterogeneity and clonality of kidney-infiltrating T cells in murine lupus nephritis

**DOI:** 10.1172/jci.insight.156048

**Published:** 2022-04-22

**Authors:** Shuchi Smita, Maria Chikina, Mark J. Shlomchik, Jeremy S. Tilstra

**Affiliations:** 1 Department of Immunology,; 2 Department of Computational and Systems Biology,; 3 Department of Medicine, and; 4 Lupus Center of Excellence, University of Pittsburgh School of Medicine, Pittsburgh, Pennsylvania, USA.

**Keywords:** Autoimmunity, Immunology, Autoimmune diseases, T cells

## Abstract

We previously found that kidney-infiltrating T cells (KITs) in murine lupus nephritis (LN) resembled dysfunctional T cells that infiltrate tumors. This unexpected finding raised the question of how to reconcile the “exhausted” phenotype of KITs with ongoing tissue destruction in LN. To address this, we performed single-cell RNA-Seq and TCR-Seq of KITs in murine lupus models. We found that CD8^+^ KITs existed first in a transitional state, before clonally expanding and evolving toward exhaustion. On the other hand, CD4^+^ KITs did not fit into current differentiation paradigms but included both hypoxic and cytotoxic subsets with a pervasive exhaustion signature. Thus, autoimmune nephritis is unlike acute pathogen immunity; rather, the kidney microenvironment suppresses T cells by progressively inducing exhausted states. Our findings suggest that LN, a chronic condition, results from slow evolution of damage caused by dysfunctional T cells and their precursors on the way to exhaustion. These findings have implications for both autoimmunity and tumor immunology.

## Introduction

Infiltration of solid organs by presumably pathogenic T cells is a common feature in many autoimmune diseases and in organ transplant rejection. These pathogenic events histologically and conceptually parallel T cell infiltration of tumors, which can lead to tumor cell destruction. However, in contrast to the well-studied tumor-infiltrating lymphocytes (TILs), tissue-infiltrating T cells mediating disease in autoimmunity have received far less attention. It is now well accepted that the tumor microenvironment functions to inhibit immune responses and induce T cell exhaustion (1–3), while in autoimmunity it is generally thought that pathogenic T cells actively destroy normal tissues unimpeded, as nonmalignant tissues lack the capacity to evolve resistance. To explore the nature of autoimmune infiltrates, we have chosen to study lupus nephritis (LN), as the kidney is a pivotal target in this autoimmune disease. T cell–dependent interstitial nephritis is the major prognostic indicator of organ failure in lupus (4, 5).

Our recent work demonstrating that kidney-infiltrating T cells (KITs) are functionally and transcriptionally exhausted (6) — much like TILs or CD8^+^ T cells after chronic viral infection — challenges 2 paradigms. First, it argues against the idea that autoimmune destruction of parenchymal tissue is a direct effector process, akin to a response to virally infected cells. Instead, some tissues may be endowed with natural self-preservation mechanisms that downregulate or suppress infiltrating T cells, which may prevent or attenuate autoimmunity. Second, it suggests that tumors do not evolve a de novo set of pathways to negatively regulate T cells. Instead, such pathways may be native to certain parenchymal tissues, and tumors may amplify them or simply naturally express them.

The discovery that kidney and perhaps other tissues can suppress infiltrating T cells raised an important question: If KITs are functionally and transcriptionally inert or exhausted, then by what mechanisms does tissue damage proceed? One hypothesis to address this question is that exhausted cells are more properly thought of as dysfunctional — rather than nonfunctional — and that they may slowly inflict tissue damage from residual effector function, much as exhausted T cells in chronic infection do serve to protect from more aggressive infection (7, 8). A second, nonexclusive explanation is that self-reactive T cells retain effector function upon arrival in the kidney, but they become progressively less functional due to the influence of the tissue microenvironment. In this view, damage by a given T cell clonotype is inflicted to a greater degree initially and then mitigated by an exhaustion program. Chronic disease could be maintained by continual recruitment of new clones that are initially more functional.

To investigate these mechanisms, it is necessary to elucidate the nature of intraparenchymal autoimmune responses more deeply. The result of such work — an understanding of the nature of damage-causing T cells — could reveal ways of specifically targeting them, for example based on their metabolic status or surface phenotype. In addition, such information could reveal disease drivers and provide tools to stratify patients — 2 important issues in lupus, which is notoriously heterogeneous in manifestation (9, 10). Further, such insights will likely apply to other target organs and disease settings.

It is, however, exceptionally challenging to address these questions in human LN, due to the scarcity of available tissue, which can only be obtained upon an extra “research pass” of a needle biopsy. Indeed, a recent consortium study, though heroic, managed to assess only about 1000 single cells by RNA-Seq over all the patients (11). Moreover, nearly all of these patients were under treatments that were designed specifically to affect these very same cells, which greatly complicates interpretation.

For this reason, here we have employed murine lupus models, which afford us access to tissue and without the complication of various treatments. We have used single-cell RNA-Seq (scRNA-Seq) to identify a surprising degree of heterogeneity among both CD4^+^ and CD8^+^ KITs, suggesting a more dynamic process of target organ damage than had been suspected. Within the CD8 compartment in particular, a large cluster of “transitional” cells was identified, which retained partial activation and effector function and was predicted to evolve into both exhausted cells as well as resident memory cells, all of which were also identified among KITs. Whereas CD4^+^ cells, intriguingly, did not fit into existing Th paradigms but rather included a cytotoxic cluster as well as demonstrating a pervasive exhausted phenotype. In addition, by using TCR-Seq, we were able to connect different clusters of KITs, delineating a compelling progressive evolution of clonotypes within the kidney. This work reveals the landscape of interactions between infiltrating T cells and the parenchyma in a lupus target organ and suggests a dynamic model for how nephritis evolves as a chronic condition. These insights could inform new strategies to interrupt the process.

## Results

### Experimental design.

**Figure 1:**
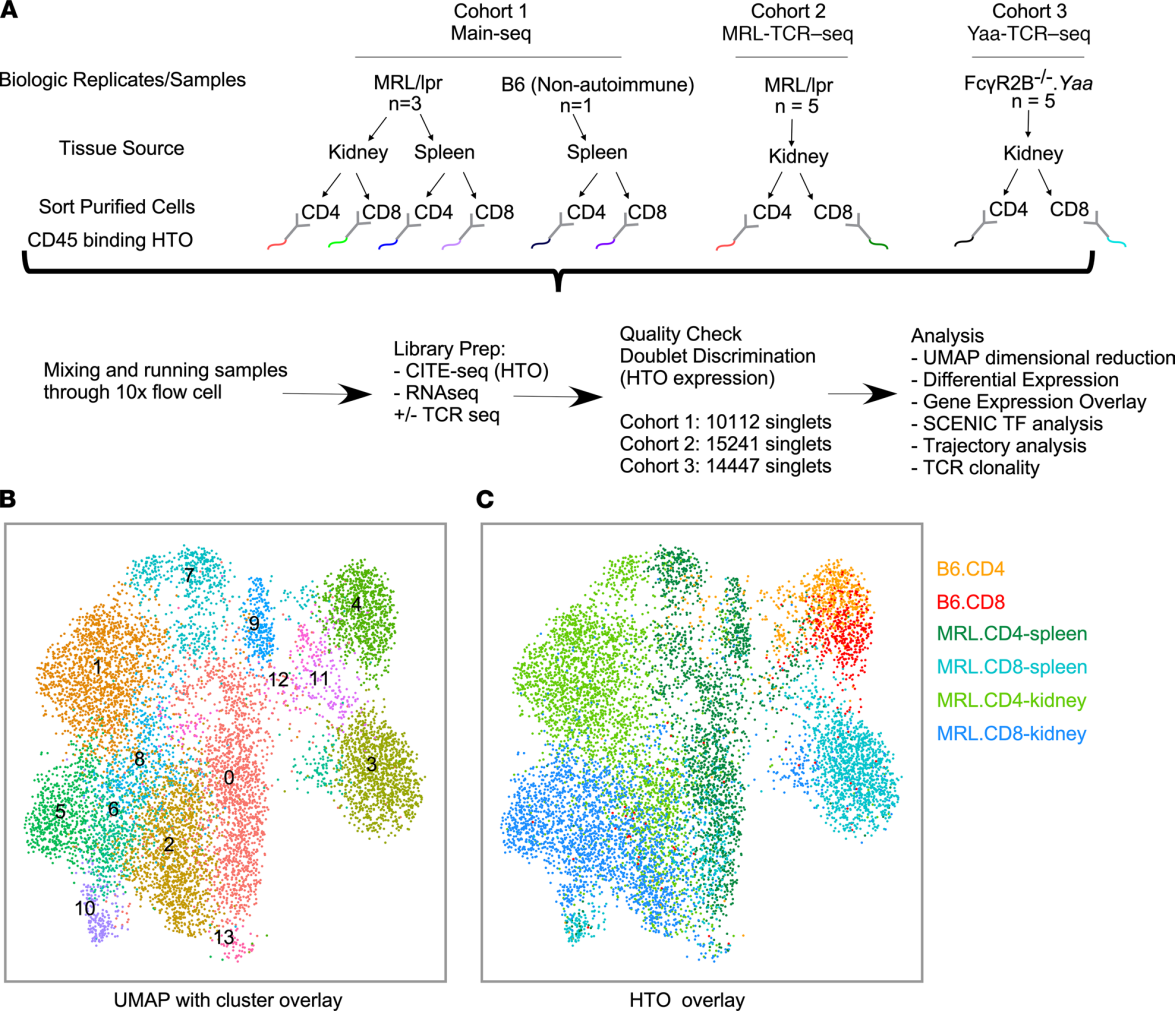
Low-resolution clustering of T cells from lupus-prone mice. (**A**) Schematic of experimental design encompassing tissue source, sorting algorithm, marking with HTOs, number of mice used in each experimental cohort, and a description of downstream analytic techniques. (**B**) UMAP of T cells from mice in Main-Seq, outlining 14 clusters. (**C**) HTO-based assignment of cell source was mapped onto the UMAP. MRL/lpr, MRL.*Fas*^lpr^; B6, C57BL/6; UMAP, uniform manifold approximation and projection; TF, transcription factor.

We generated 3 experimental cohorts to address T cell reprogramming after tissue infiltration in murine LN using the 10x Genomics scRNA-Seq platform and hashtag oligonucleotide (HTO) deconvolution technology (Figure 1A). In the initial cohort (Main-Seq), we compared splenic T cells and KITs from aged lupus-prone nephritic MRL/lpr mice, as well as “naive” T cells from non-lupus-prone B6 mice. In the second cohort, we focused on KITs from MRL/lpr mice and in addition assessed the KIT TCR repertoire (MRL-TCR–Seq). Finally, in the third cohort, we completed a similar KIT analysis as in cohort 2 using the FcγR2B^–/–^.*Yaa* model of LN (Yaa-TCR–Seq).

### T cells cluster based on origin, autoreactive status, and CD4/8 phenotype.

Low-resolution transcriptome-based clustering of all T cells in the Main-Seq cohort, using Seurat (12), identified 14 clusters (Figure 1B). HTO deconvolution demarcated cells based on T cell type (i.e., CD4 and CD8), organ of origin (kidney vs. spleen), and potential autoreactive status (B6 vs. lupus-prone mice, Figure 1C). All 3 MRL/lpr mice contributed to each defined population, with no population being driven by a specific donor (Supplemental Figure 1; supplemental material available online with this article; https://doi.org/10.1172/jci.insight.156048DS1). While CD4^+^ and CD8^+^ T cells from non-autoimmune mice were relatively uniform, CD4^+^ and CD8^+^ T cells from diseased mice displayed remarkable heterogeneity (Figure 1, B and C).

### Heterogeneity and potential functions of CD4^+^ T cells in murine lupus.

**Figure 2:**
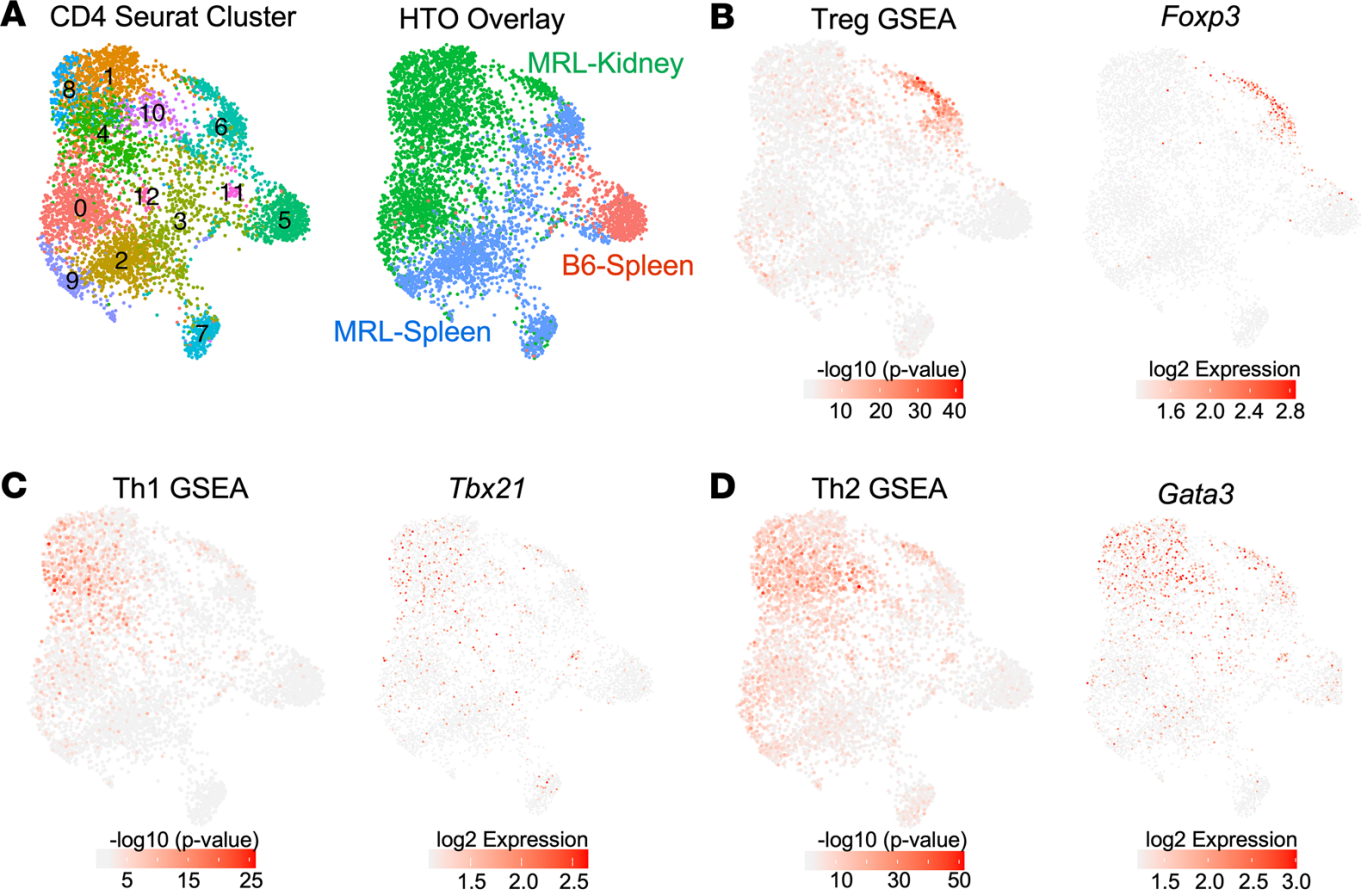
High-resolution clustering of CD4^+^ T cells uncovers unique transcriptional programs in KITs. (**A**) UMAP of CD4^+^ T cells from Main-Seq outlining 13 clusters, with the right panel exhibiting assignment of cell source as determined by HTO. (**B**–**D**) Gene set enrichment analysis (GSEA) performed in each cell by Wilcoxon’s test (–log_10_ [*P* value]) using published reference gene signatures (Supplemental Table 1) and related TF expression were overlaid onto the UMAP to identify CD4 phenotypes. This included (**B**) Treg gene signature and *Foxp3* expression, (**C**) Th1 signature and *Tbx21*/Tbet expression, and (**D**) Th2 signature and *Gata3* expression.

High-resolution clustering yielded 13 CD4^+^ T cell clusters (Figure 2A). HTO-directed deconvolution highlighted separation between kidney-derived and spleen-derived T cells from MRL/lpr mice, while CD4^+^ T cells from B6 mice fell predominantly into 2 clusters (5 and 11) (Figure 2A, right panel). By identifying the most differentially expressed genes (DEGs) in each cluster (Supplemental Figure 2), we attempted to assign specific phenotypic subtypes to each CD4^+^ cluster. Using this approach, only 2 of the CD4^+^ T cell clusters were easily matched to conventional T cell phenotypes: cluster 6 represented Tregs, while cluster 12 exhibited a type I IFN signature (IFN^hi^).

We then examined TF expression and canonical T cell gene expression signature enrichment overlays for well-described CD4^+^ T cell phenotypes, including Treg, Th1, Th2, T follicular helper (Tfh), and Th17 (see Supplemental Table 1 for gene lists) (13–23). As expected, the presumed Treg cluster exhibited strong Treg-associated gene signatures and expressed *Foxp3* (Figure 2B). The Th1 signature was associated with clusters 1, 4, and 8, and *Tbet* was expressed in a similar distribution (Figure 2C). A stronger Th2 gene signature and *Gata3* expression were also observed in the kidney-predominant clusters 1, 4, 8, and 10, suggesting some coexpression of Th1 and Th2 phenotype with no clear delineation between the 2 lineages (Figure 2, C and D). Several reports have suggested a role for Th17 in LN; however, similar to the findings in the human nephritis samples the Accelerating Medicines Partnership in SLE network (AMP consortium) examined (11), no significant Th17 profile was observed by gene signature or *Rorc* expression in any compartment (Supplemental Figure 3, A and B). Few cells exhibited either the Tfh-associated gene signature or *Bcl6* expression, consistent with reports of few germinal centers in MRL/lpr spleens (24) (Supplemental Figure 3C). Finally, cluster 7 probably represented a central memory T cell (T_CM_) compartment, and cluster 5 represented naive T cells (Supplemental Figure 3, D and E).

**Figure 3:**
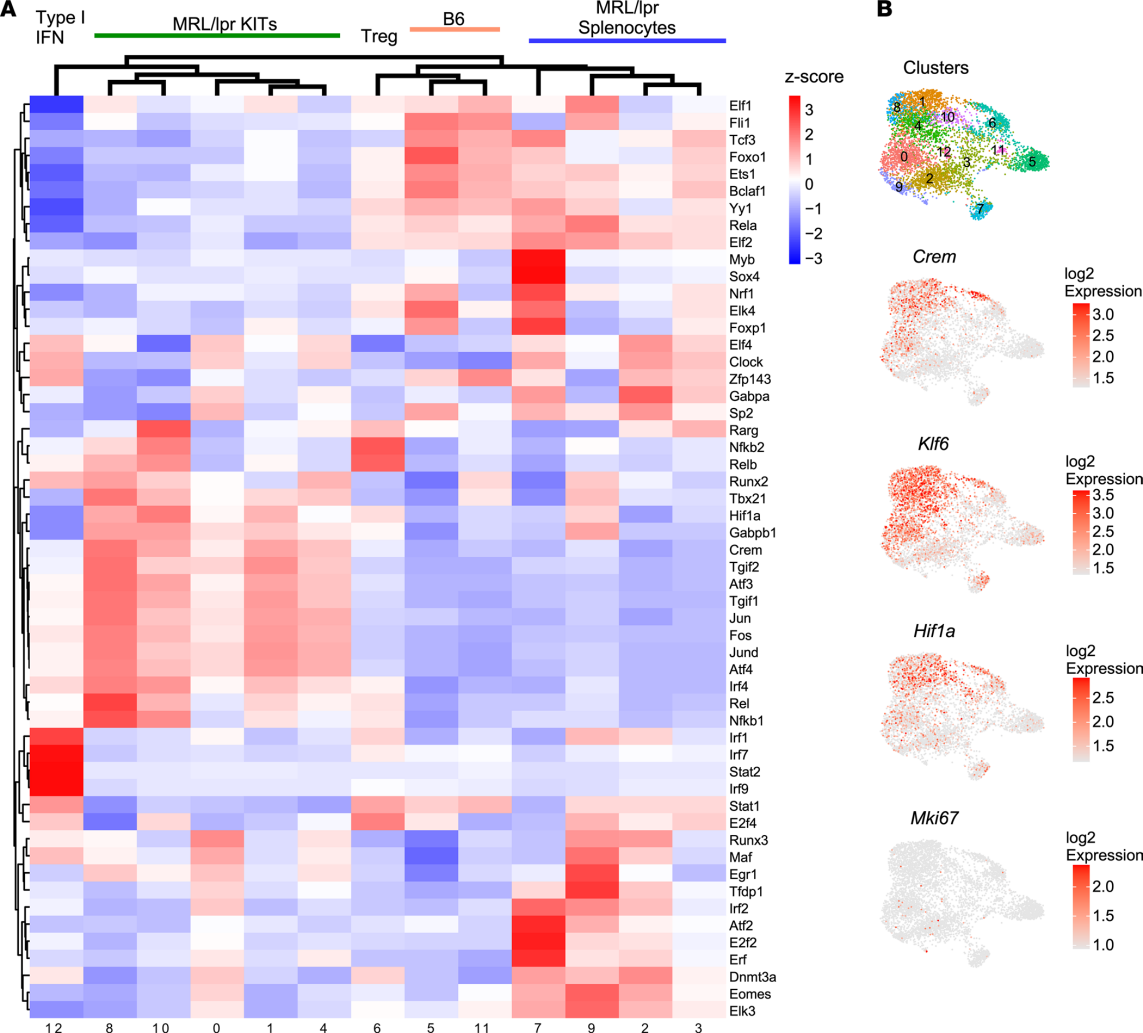
TF analysis suggests overarching transcriptional regulation of infiltrating CD4^+^ T cell clusters. (**A**) Heatmap representing *z*-scored regulon activity of top TFs inferred by SCENIC and association with CD4^+^ T cell clusters. (**B**) Expression of selected TFs overlaid onto the CD4 UMAP as depicted in Figure 2.

**Figure 4:**
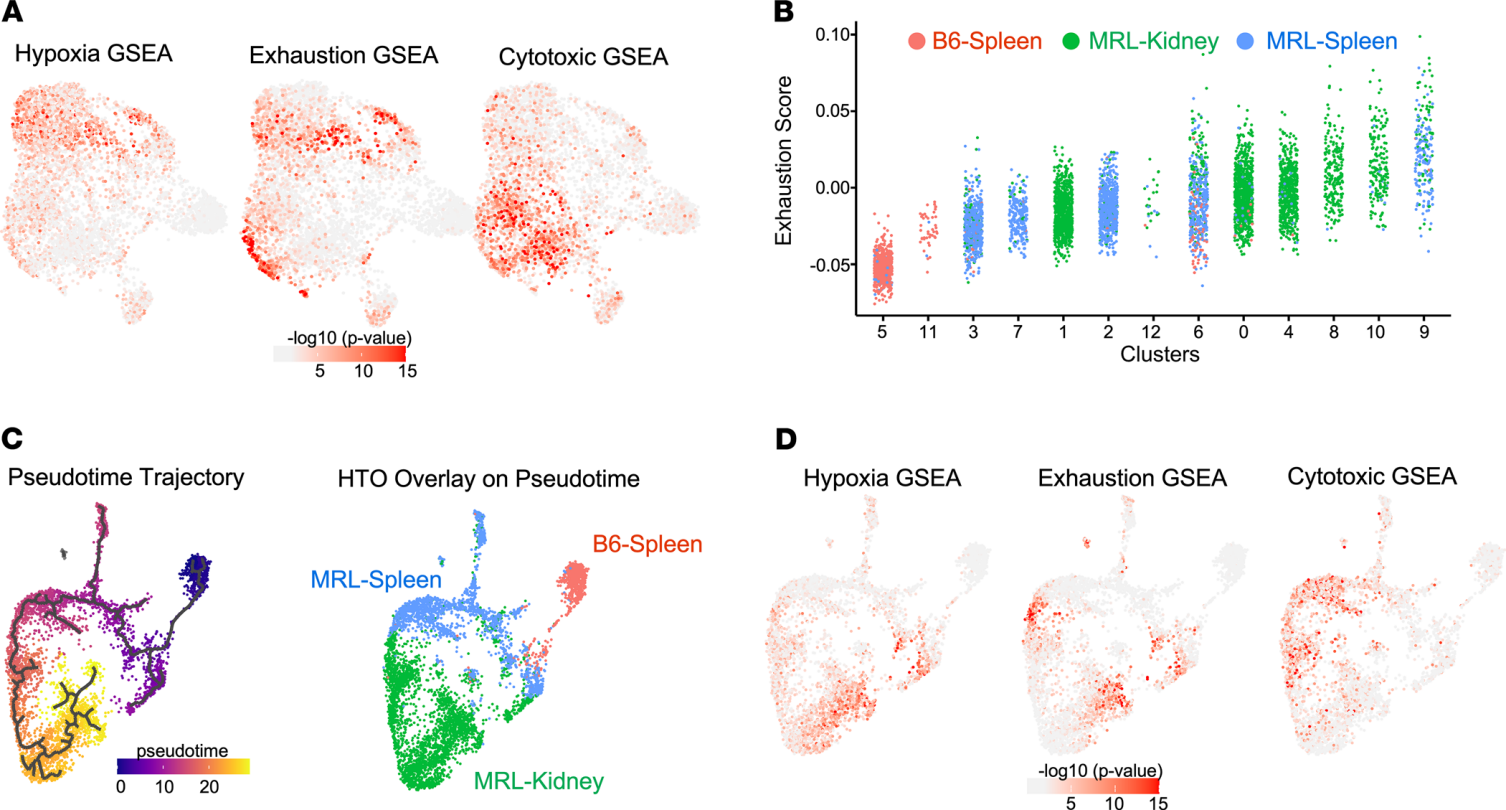
CD4^+^ KITs exhibit a progressive transcriptional phenotype from cytotoxicity to hypoxia/dysfunction through pseudotime. GSEA performed in each cell by Wilcoxon’s test (–log_10_ [*P* value]) using published reference genes signature (Supplemental Table 1) overlaid onto the UMAP from Figure 2A. (**A**) Hypoxia signature, exhaustion signature, and cytotoxic CD4 signature. (**B**) Dot plots show the distribution of exhaustion score in each CD4^+^ T cell, grouped by cluster number. Dots are colored according to the source of cells they represent. Statistics were calculated by Kruskal-Wallis rank test. (**C**) Monocle trajectory mapping of CD4^+^ KITs wherein time 0 (dark purple) represents lineage origination with progression to most differentiated (yellow), with cell source mapping. (**D**) Gene signature mapping for the indicated signatures as defined in **A**.

We identified several TFs whose motifs were enriched among DEGs of given clusters using the SCENIC algorithm. Although the type I IFN cluster (cluster 12) shows evidence of IRF1, IRF7, IRF9, and STAT2 regulation (25) (Figure 3A), in general we did not find unique TF associations within specific clusters. However, hierarchical cluster analysis of the TF suggested transcriptional organization by organ of origin or presence of autoimmunity. There was a kidney-specific influence, in that numerous TF binding motifs (including HIF1α, TGIF2, CREM, JUN, FOS) were enriched in the majority of KITs, irrespective of cluster; several of these TFs were also themselves transcriptionally upregulated, including *Crem* and *Hif1α*, which have previously been associated with tissue residency and hypoxia, respectively (18, 26, 27) (Figure 3B). In accordance with the *Hif1α* expression and transcriptional activity, recent work described a hypoxia gene expression profile in bulk RNA-Seq of KITs isolated from MRL/lpr mice (18). Similarly, we observed that a portion of KITs expressed a hypoxia profile, particularly in clusters 1, 4, 8, and 10 and the KIT Treg population (Figure 4A).

Given the findings from our prior work suggesting KIT exhaustion, we assessed the exhaustion signature (6), which appeared enriched among CD4^+^ KITs in total (Figure 4, A and B). Exhaustion signatures in multiple clusters were higher than the baseline defined by CD4^+^ B6 splenic T cells, with clusters 0, 4, 8, 10, and 9 exhibiting the highest score (Figure 4B). These clusters were composed mainly of KITs, with the exception of cluster 9, which consisted of both KITs and splenic T cells from MRL/lpr mice; however, even in cluster 9, the more exhausted cells were kidney derived (Figure 4B). Hence, despite the heterogeneity of CD4^+^ KITs, exhaustion-associated gene expression appears in the transcriptomes of multiple clusters of T cells.

As cytotoxic CD4^+^ T cells have been reported in aging, (13) tumor infiltrates (28), and systemic sclerosis (29), we assessed whether a cytotoxic gene signature was observed in KITs. Indeed, a portion of both splenic and kidney-derived T cells from MRL/lpr mice, encompassing clusters 0, 2, and 9, expressed a cytotoxic gene signature (Figure 4A). Interestingly, KIT clusters generally expressed either a hypoxic profile or a cytotoxic gene signature but not both.

### Trajectory analysis identifies developmental progression of CD4^+^ T cells.

Based on the analysis of cluster gene signatures, we hypothesized that there is a progression from naive to peripheral effector/cytotoxic T cells, which then invade the kidney where they first express a cytotoxic program, followed by a hypoxia response accompanied by exhaustion/dysfunction. To evaluate this model, we created a lineage progression analysis using Monocle3, which integrates temporal gene expression (Figure 4C). HTO overlays on the Monocle3 pseudotime trajectory (Figure 4C) indicated that lineage origin occurs with the B6 naive cluster; the program then postulated early branching of the Treg cluster, along with progression through activated lupus-prone splenic cells, and finally to kidney-infiltrating cells. In this model, it appears that naive T cells enter the “autoimmune/cytotoxic phase” just prior to or in parallel with kidney infiltration, as hypothesized, and finally progress to a hypoxic/dysfunctional state (Figure 4D).

### KIT Tregs express tissue-resident markers.

**Figure 5:**
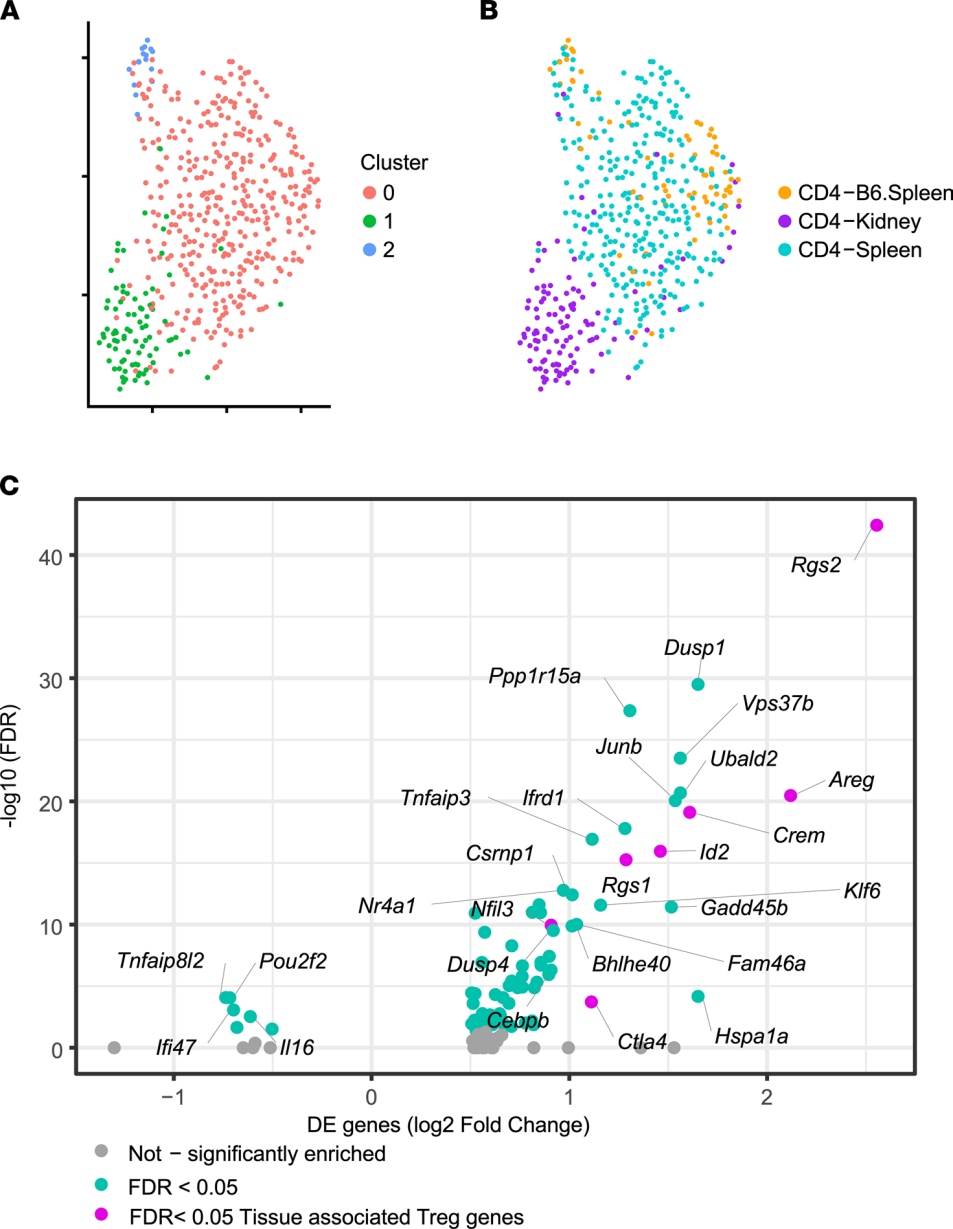
Kidney-infiltrating Tregs exhibit features of tissue reprogramming. (**A**) High-resolution reclustering of Tregs identified as cluster 6 in Figure 2 (Main-Seq) identifies 3 unique clusters as illustrated by color-coding. (**B**) HTO-based identification of splenic (B6 and MRL/lpr) and KIT Tregs. (**C**) Volcano plot shows top significant (FDR < 0.05) DEGs that are upregulated or downregulated in KITs compared with splenic MRL/lpr Tregs, with genes in pink having been previously associated with tissue-resident Tregs (30).

To assess the independent effect of tissue reprogramming on T cell phenotype, we more closely evaluated the Treg population (cluster 6), as this cluster was composed of cells from all 3 origins. Upon reclustering, KIT Tregs mapped separately from the splenic Tregs of either B6 or autoimmune origin (Figure 5, A and B). A volcano plot (Figure 5C) revealed a number of DEGs between peripheral and kidney Tregs, mostly upregulated in the kidney, suggesting that tissue residence induced an additional transcriptional program. These DEGs included *Crem* and *Klf6*, TFs previously associated with CD4^+^ KIT phenotypes in our TF mapping (Figure 3B). Interestingly, genes previously identified in tissue-resident Tregs when compared to lymphoid Tregs were similarly transcriptionally upregulated in KIT Tregs, including *Crem*, *Rgs1*, *Rgs2*, *Id2*, *Ctla4*, and *Areg* (Figure 5C, highlighted in pink) (30).

### CD8^+^ KITs exhibit heterogeneity after tissue infiltration.

**Figure 6:**
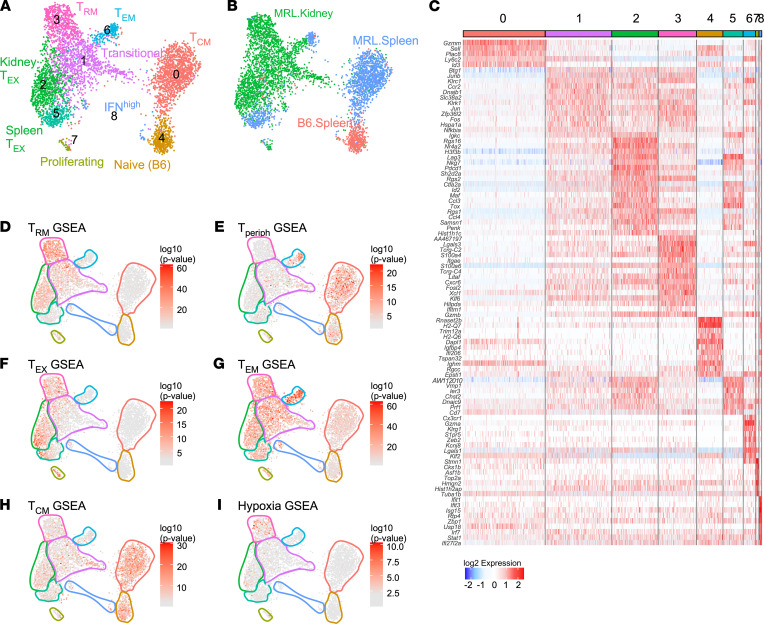
High-resolution clustering of CD8^+^ T cells identifies unique functional phenotypes. (**A**) UMAP of CD8^+^ T cells from Main-Seq delineating 9 clusters. (**B**) UMAP with overlay exhibiting assignment of cell source as determined by HTO. (**C**) Heatmap shows top significant (FDR < 0.01) DEGs associated with each CD8^+^ cluster and their expression at single-cell level in columns. (**D**–**I**) GSEA performed in each cell by Wilcoxon’s test (–log_10_ [*P* value]) using published reference gene signatures (Supplemental Table 1), overlaid onto the UMAP to identify CD8 phenotypes. Clusters are outlined as per **A**. This included gene signatures for (**D**) resident memory (T_RM_), (**E**) circulating/lymphoid (T_periph_), (**F**) exhaustion (T_EX_), (**G**) effector memory (T_EM_), (**H**) central memory (T_CM_), and (**I**) hypoxia.

CD8^+^ T cells (from Main-Seq) were analyzed as done for the CD4^+^ population, yielding 9 clusters that resolved based on HTO (Figure 6, A and B). We could identify most of these clusters by virtue of the DEGs in each (Figure 6C). The majority of splenic T cells were clustered to the right side of the UMAP, with cluster 4 being predominantly composed of B6 splenic T cells and cluster 0 representing the majority of MRL/lpr splenic T cells and suggesting a T_CM_ phenotype.

Autoimmune splenic T cells were also represented in clusters 6 and 5, which comprise T effector memory (T_EM_) and peripherally exhausted cells, respectively. CD8^+^ KITs from MRL/lpr mice were found in several unique clusters. Cluster 2 contained an exhausted T cell population (T_EX_). Cluster 3 consisted of a resident memory T cell population (T_RM_). Cluster 6 was a T_EM_ cluster, made up of approximately 50% KITs and 50% splenic T cells. Cluster 1 was the largest KIT cluster. It appeared to represent a transitional state, as cells in this cluster expressed a mixture of genes indicative of T_EX_, T_RM_, and T_EM_. The 2 smallest clusters were dividing cells (cluster 7), and IFN^hi^ T cells (cluster 8), with cells from multiple sources.

To confirm clusters’ putative identities, reference gene signatures (Supplemental Table 1) were overlaid on the UMAP plots (Figure 6, D–I). The resident memory signature mapped most strongly to the T_RM_ and KIT T_EX_ clusters (Figure 6D). Reciprocally, the peripheral gene signature was associated with splenic derived T cells — particularly the naive, the T_CM_, and splenic portion of the T_EM_ clusters (Figure 6E); however, the peripheral T_EX_ cluster exhibited neither a T_RM_ or a peripheral signature. As expected, the CD8^+^ KIT population as a whole manifested a robust “exhausted” signature (6). Among KITs, the exhaustion signature was most associated with clusters 2 and 3 and to a lesser extent 1, which represent the KIT T_EX_, T_RM_, and transitional populations, respectively (Figure 6F). Reference gene UMAP overlays for T_EM_ and T_CM_ transcriptional signatures confirmed our initial classifications of these clusters (Figure 6, G and H).

### Unique TF profile associated with T cell infiltration and exhaustion.

**Figure 7:**
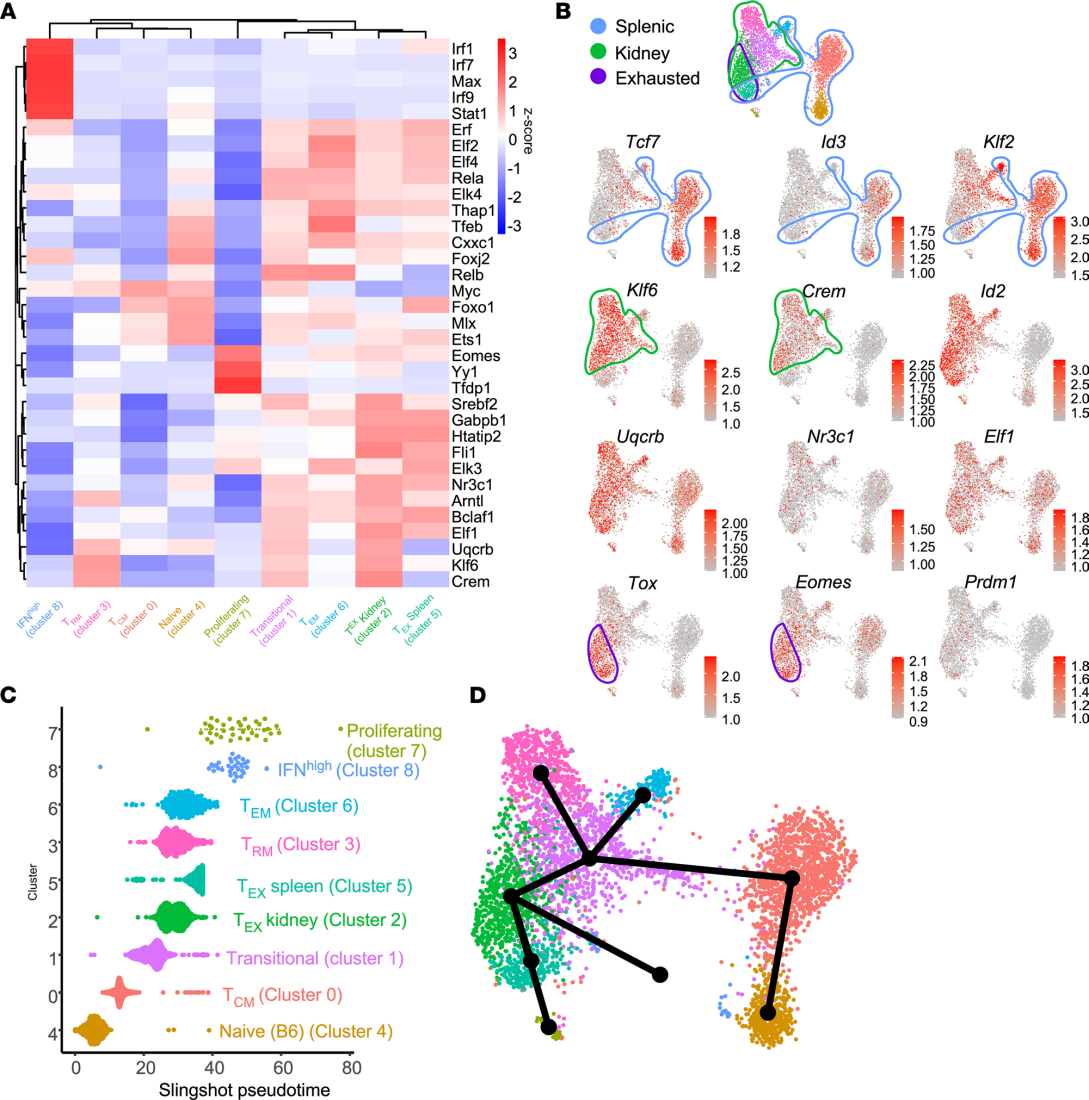
TF and lineage progression analysis of CD8^+^ KITs. (**A**) Heatmap represents *z*-scored regulon activity of top TFs inferred by SCENIC (rows) and association with CD8^+^ T cell clusters (columns). (**B**) Representative TFs were mapped onto CD8 UMAPs; TF selection was based on known regulatory functions or due to identification via SCENIC analysis. Outlines highlight spleen-derived, kidney-infiltrating, and exhausted cells, with dot red color intensity representing log_2_ expression. (**C**) CD8^+^ T cells grouped into 9 distinct clusters and ordered by Slingshot pseudotime trajectory. (**D**) Slingshot lineage overlay on CD8^+^ T cell UMAP.

We further performed TF motif enrichment for the CD8^+^ T cell clusters using SCENIC (Figure 7A). As expected, IRF1, IRF7, IRF9, and STAT1 were identified as transcriptional drivers of the IFN^hi^ cluster. Similar to the CD4^+^ compartment, several motifs were enriched in all CD8^+^ KITs (UQCRB, CREM, and KLF6), while other TFs were cluster specific; for example, in the T_EX_ clusters (2 and 5), motifs for NR3C1, HTATIP2, and ELF1 were enriched.

Not all TFs can be mapped by SCENIC. To provide an alternative approach, we overlaid expression of multiple TFs on the UMAP plots (Figure 7B). Expression of *Crem* and to a lesser extent *Klf6*, was associated with KITs, while *Uqcrb* was not, exposing some discordance between TF expression and predicted TF gene regulation. Expression of both *Tox* and *Eomes*, which are regulators of T cell exhaustion (31–33), are correlated with the T_EX_ clusters 2 and 5, further validating their identity. Notably, *Tcf7*, which is strongly expressed in splenic T cells, was also observed in transitional cells, with expression generally extinguished in T_EX_ and T_RM_ populations. A pattern similar to *Tcf7* was seen with *Klf2*, whereas *Klf6* and *Id2* had reciprocal patterns.

### Trajectory analysis identifies lineage progression of CD8^+^ T cells and a distinct transitional population.

Based on the fact that cluster 1 was at the center of the clearly distinguishable T_EX_, T_RM_, and T_EM_ clusters, and that cluster 1 displayed intermediate expression of several gene signatures (T_RM_, T_EX_, T_periph_) and TFs (*Crem*, *Tcf7*, *Tox*, *Klf2*, and *Klf6*) (Figure 6, D–F, and Figure 7B), we hypothesized that cluster 1 represents a transitional population that is in the process of differentiating from a peripheral phenotype to any one of the 3 more differentiated terminal destinations. Interestingly, a potentially similar transitional cell has been identified in tumor models in which cells also exhibit an exhausted or effector phenotype (34). To evaluate this hypothesis, we performed computational lineage progression analysis (Figure 7, C and D). This analysis delineated a progression from naive to memory cells, followed by kidney tissue invasion, where cells initially manifest a “transitional” phenotype. From there, the algorithm predicted cells developing into a T_EM_ (cluster 6), T_EX_ (cluster 2), or T_RM_ (cluster 3) phenotype.

### Clonal expansion and proliferation are associated with an exhausted phenotype in KITs.

If the model that autoreactive T cells enter the kidney and then become exhausted as part of a self-preservation mechanism is correct, we would expect the transitional and exhausted clusters to harbor clonally expanded T cells, with some of the clones spanning both clusters, suggesting dynamic progression. To test this, we performed TCR-Seq of single cells, which allowed clonal lineage tracking among clusters in both genetic models of murine LN (MRL-TCR–Seq and Yaa-TCR–Seq, respectively).

**Figure 8:**
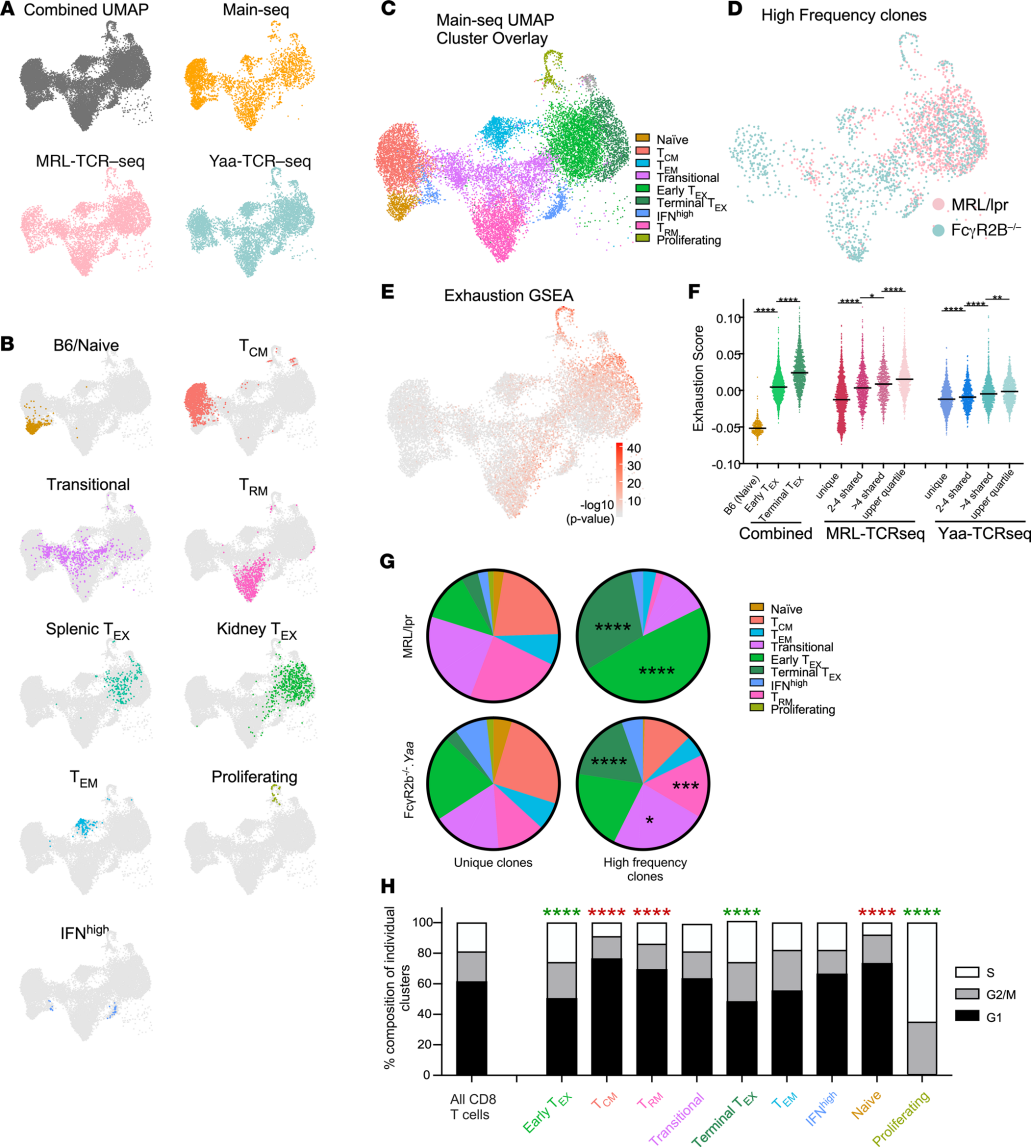
CD8^+^ KITs are clonally expanded, with clones and proliferation spanning the exhausted and transitional compartments. (**A**) UMAP of all 3 scRNA-Seq cohorts (see Figure 1 for cohorts), integrated using Harmony, followed by projection of individual cohorts onto this “combined UMAP.” (**B**) Main-Seq–defined CD8^+^ clusters labeled by cluster name (Figure 6), mapped onto the combined UMAP. (**C**) Combined UMAP with all putative clusters as outlined in **B**. (**D**) High-frequency clones (defined as clones representing the top quartile of expressed TCRs) from each cohort are mapped onto the combined UMAP. (**E**) Exhaustion gene set enrichment calculated using Wilcoxon’s test overlaid onto the combined UMAP. (**F**) Dot plots represent exhaustion scores for cells grouped based on clonal frequency among MRL/lpr and FcγR2B^–/–^.*Yaa* KITs, with exhaustion scores for B6 naive, early T_EX_, and terminal T_EX_ shown at left for reference (*P = 0.05, **P < 0.01, ****P < 0.0001 as determined by 1-way ANOVA with Tukey’s test for multiple comparisons). Horizontal bars represent medians. (**G**) Pie charts represent the relative cluster distribution of unique TCR T cells as compared with high-frequency TCRs from MRL/lpr (top) and FcγR2B^–/–^.*Yaa* (bottom) models with the relative distribution of these clones within the putative T cell clusters as defined in **B** and **C**. (**H**) Cell cycle state was assessed over all cells for each cluster using GSEA for genes indicative of G1, G2/M, and S phases. Proliferative potential was analyzed for enrichment of G2/M and S phase genes, comparing individual clusters with the total T cell population. (**G** and **H**) Data were analyzed using χ^2^ analysis and corrected for multiple comparisons for 9 comparison groups (*P = 0.05, ***P < 0.005, ****P < 0.0001). Green indicates enrichment of G2/M/S phase genes, and red indicates enrichment of G1 genes.

Transcriptional data from all 3 cohorts were merged into a single UMAP using Harmony (Figure 8A). Using GSEA and mapping of Main-Seq cohort clusters, we identified, in the combined UMAP plot, clusters with exhausted, effector, resident memory, and transitional phenotypes (Figure 8, B and C). Despite examining 2 unique spontaneous nephritis models with variable levels of histologic disease (Supplemental Figure 4 and Supplemental Table 2), the composition of the T cell clusters of KITs was quite similar (Supplemental Figure 5 and Supplemental Figure 6, A and B), When evaluating clusters by model, only the IFN^hi^ cluster was differentially represented, being increased among the FcγR2B^–/–^.*Yaa* KITs. This represented one of the small clusters, and the increase in IFN^hi^ cells was expected based on prior data (35). To assess whether mouse-to-mouse variability in cluster distribution, particularly with regard to the exhausted population, was correlated with histologic status, we performed linear regression analysis of glomerulonephritis as well as interstitial nephritis score as compared to percentage of cells in the exhausted clusters and found no significant associations (Supplemental Figure 6C). By histologic analysis (Supplemental Figure 4 and Supplemental Table 2) MRL/lpr mouse 5 had the most severe disease and the highest terminal exhaustion score; however, the remainder of the mice with variable levels of disease did not offer any additional correlation between histologic disease score and CD8^+^ T cell phenotype. Overall, these 2 genetically distinct murine lupus models had very similar cluster representation among their KITs, indicating at a single-cell level that the processes underlying interstitial nephritis were not model specific (Supplemental Figures 5 and 6).

We identified expanded T cell clones in each of the mice evaluated, consistent with previous reports in patients with systemic lupus erythematosus (SLE) (36–38). High-frequency CD8 clones (top quartile) were defined as a TCR shared between ≥13 T cells (or a frequency of ~1%) in MRL-TCR–Seq and between ≥40 T cells (or a frequency of ~3%) in Yaa-TCR–Seq (Figure 8D). In the combined UMAP, 2 distinct clusters of exhausted cells were observed. The rightmost cluster (Figure 8E) exhibited a significantly (*P* < 0.0001) higher exhaustion gene signature, which we denote as a terminally exhausted cluster (Figure 8F). In the MRL/lpr model the great majority of the high-frequency clones mapped to the exhausted clusters (comprising about 79% of all high-frequency clones), which is a significant enrichment compared with T cells expressing unique TCRs (*P* < 0.0001, Figure 8, D and G). There was less robust expansion of clonal T cells in the FcγR2B^–/–^.*Yaa* model, with 34.4% of high-frequency clones mapping to the 2 exhausted clusters, compared with an expected 23.1% of all T cells (*P* < 0.0001). Notably, there was significant clonal expansion observed in the terminal exhaustion cluster: among T cells expressing unique TCRs, the terminal exhaustion cluster comprised only 3.12%; whereas among high-frequency clones, the terminal exhaustion cluster comprised 17.3% of cells (*P* < 0.0001). The 2 other clusters that displayed expansion of the high-frequency clones in the FcγR2B^–/–^.*Yaa* mice were T_RM_ cells and the transitional group. The transitional cluster represented 17.1% of cells with unique TCRs compared with 23.9% of the high-frequency clones (*P* < 0.05, Figure 8G). Importantly, and consistent with TIL data (39), higher clonal abundance was progressively correlated with higher exhaustion score as measured for high-frequency, moderate-frequency (>4 T cells sharing a TCR), shared (2–4 cells), and unique clones (Figure 8F).

Expectedly, there was very little overlap among TCRs between mice. None of the top clones showed overlap between the MRL/lpr and FcγR2B^–/–^.*Yaa* models. When examining the top 10 clones from each model, only the top clones showed any overlap. In each case nearly all the TCRs sharing the same CDR3 regions arose from a single mouse, with 1 clone arising from a second mouse in the MRL/lpr cohort. Although 3 mice in the FcγR2B^–/–^.*Yaa* cohort expressed the top clonal sequence, 168 T cells arose from 1 mouse while only 1 each arose from the 2 other mice (data not shown). We are uncertain whether these are true, independent instances of the same TCR sequence or are the result of rare demultiplexing errors arising from unfiltered doublets.

Plotting the distribution of the top 20 highest frequency clones (Supplemental Figure 7) allowed us to evaluate how large clones span clusters, which in turn directly links the evolution of these clones over the cluster phenotypes. In every high-frequency MRL/lpr clone, members were found in the transitional, T_EX_, and terminal T_EX_ clusters. In the Yaa model, the majority of the highest frequency clones were also represented in transitional as well as more terminally differentiated clusters, including both T_EX_ and T_RM_ clusters. These clones provide direct evidence that progeny of the same cell transition between differentiation states within the kidney. Taken together, these data support the trajectory analysis (Figure 7D), with clonal expansion in the transitional compartment followed by terminal differentiation.

### Proliferation in exhausted clusters.

Expanded clones in exhausted clusters could result from continued proliferation after differentiation into those clusters or could be nondividing terminal differentiation products of their dividing transitional precursors. To distinguish these possibilities, we quantified the cell cycle state of each cluster using a score for expression of gene sets indicative of cell cycle phase. As expected, the proliferative cluster (Figure 8B) was enriched in S phase and to a lesser extent G2/M genes. The KIT exhausted clusters had high and significant S phase and G2/M scores in both strains of mice (Figure 8H). In contrast, the naive, T_RM_, and T_CM_ clusters exhibited significant enriched expression of G1 phase genes, indicative of resting, stable populations. This supports the concept that exhausted cells do continue to proliferate and that clonal expansion can occur at least in part after exhausted differentiation, suggesting that exhausted cells are sensing TCR signals and may contribute directly to pathogenesis.

## Discussion

We previously reported that KITs in 3 different murine lupus models exhibited functional and transcriptional “exhaustion,” with characteristics that paralleled TILs in progressing tumors (6, 14, 40). Yet, nephritis in both lupus-prone mice and patients with SLE results in the ultimate destruction of tissue and loss of organ function. The dynamic process that reconciles these 2 findings, and that describes how autoimmune renal destruction occurs and at the same time may be restrained via exhaustion and other mechanisms, has not been elucidated. Here, we have used scRNA-Seq and TCR-Seq to reveal marked heterogeneity among KITs and a likely progressive pathway that connects initially activated and potentially destructive CD4^+^ and CD8^+^ T cell clones to exhausted populations of cells. Together, these studies provide insights into the pathogenesis of LN and potentially actionable information for novel treatment strategies.

A major goal of this work was to define which populations might be contributing to damage in LN. While numerous cell populations have been implicated in the pathogenesis of LN, including dendritic cells, macrophages, B cells, and neutrophils (41), in our prior work we determined that T cells exhibited the largest expansion in nephritic mice compared with non-nephritic mice (6). They also exhibited unique functional features, and thus we selected this population for further analysis. However, it is likely that numerous other cell populations likely contribute to disease observed in both mice and humans. In the CD4^+^ compartment, clustering based on gene expression did not reveal classical CD4^+^ T cell differentiation states, including Th1, Th2, or Th17 cells; rather, several clusters were associated with hybrid transcriptional features of both Th1 and Th2 cells (Figure 2). This would argue that the classic Th paradigm does not necessarily hold in systems of complex immune activation within tissues, as has also been observed by others (11, 42). Th1/Th2 hybrid T cells may engender a self-limited response that serves to limit excessive immunopathology (43, 44).

Trajectory analysis and gene expression mapping suggested the following model for the evolution of CD4^+^ KITs: cytotoxic self-reactive CD4^+^ T cells that arise in the periphery infiltrate the kidney, and after initiation of inflammation and transient organ damage, give rise to a more hypoxic and exhausted/dysfunctional T cell population, resulting in smoldering inflammation. The hypoxia-induced suppression and dysfunctional status of such T cells has been well documented, supporting this model (3, 45); in fact recent work suggests that hypoxia directly induces T cell exhaustion (46) consistent with our findings in this work. The proposed initial activation in the periphery predicts that some antigens recognized by CD4^+^ T cells in the periphery may also be presented in the kidney and/or that kidney-specific antigens are presented in the periphery subsequent to initial renal damage and release.

Among CD8^+^ KITs, we identified several populations, including T_CM_, T_EM_, T_RM_, IFN^hi^, and T_EX_, as well as a transitional population. A specific focus, given our prior findings and TIL literature (2, 6, 14), was the exhausted cell cluster. T_EX_ cells are associated with the TFs *Tox* and *Eomes*, documented mediators of exhaustion (31–33). Furthermore, the glucocorticoid receptor NR3C1, which was recently identified as a mechanism of promoting T cell dysfunction in tumors (47), is a potential transcriptional driver of the T_EX_ phenotype in this system. The role of the transitional population, which we elucidate here for the first time to our knowledge, may be key in understanding the dynamic process that occurs in the kidney as disease is established. Trajectory mapping placed these cells at the intersection of autoreactive peripheral T cells and terminally differentiated KITs, which would infiltrate the kidney in the context of pseudotime.

In the MRL/lpr model, clonally expanded TCRs spanned the transitional and T_EX_ populations, indicating dynamic movement between the clusters, as predicted by the trajectory model. In the FcγR2B^–/–^.*Yaa* model, clones were even more expanded; though prominent expansion was seen in the transitional and terminally exhausted clusters, large clones spanned several compartments, suggesting infiltration followed by proliferation and differentiation into multiple terminally differentiated phenotypes. Overall, the cluster distributions between the 2 models were quite similar, suggesting common themes that likely apply to human LN and other forms of autoimmune tissue infiltration. The greater extent of clonal expansion and sharing among clusters seen in the FcγR2B^–/–^.*Yaa* model may represent different extent or quality of disease, reflecting subtypes of murine LN, akin to different types of LN seen in patients. In the FcγR2B^–/–^.*Yaa* model, we noted an increased number of clonally expanded T_RM_ cells; however, the role of T_RM_ in promoting disease is unclear. While the function of these cells in barrier tissue is generally felt to provide a rapid antigen-specific response at initial pathogen encounter, the role in nonbarrier tissue may be quite different. As there are shared TCR clones between exhausted and resident memory populations, this suggests that these may be alternative differentiation pathways for T cells, and it is possible that T_RM_ may contribute to some of the chronic damage response observed in LN. Resolution of this question will await tools to specifically target either T_RM_ or T_EX_ in tissues of lupus-prone mice.

Notably, 2 recent human studies examining T cells in the tumor microenvironment (34, 39) revealed infiltrates that parallel those in the LN models. Li et al. (34) described naive, memory, transitional, and dysfunctional CD8^+^ T cells in tumor infiltrates. Much like we observed in KITs, they also observed clonal overlap between transitional and dysfunctional T cells but to a lesser degree in the cytotoxic populations (34). Further, in both KITs (herein) and TILs (34), proliferative capacity was greater among the exhausted/dysfunctional T cells, suggesting that TIL behavior may reflect enhancement of the natural processes that evolved for organ preservation in the face of autoimmunity.

At each stage of progression from activation and damage toward either exhaustion or resident memory status, there may be opportunities to intervene based on specific cellular states. Further studies to better understand the factors that sustain cells or that could inhibit cells at each given stage are needed, as are insights into the signals that promote the progression along this pathway. The progressive pathway we have identified could also be a framework for understanding genetic susceptibility loci, some of which might impinge on, for example, the likelihood that KITs can be induced toward exhaustion by the renal environment. Such genetic variants could be expressed in either the T cells or the target tissues (48).

Together these concepts raise the question of how tissues reprogram activated infiltrating T cells and what the hallmarks are of such reprogramming. In this regard, the TFs *Crem*, *Id2*, and *Klf6* were identified in all 3 KIT populations (CD4, CD8, Treg). *Crem* is a transcriptional repressor that suppresses IL-2 transcription and is associated with anergic T cells (49). *Klf6* is thought to promote T cell quiescence, and in a recent study its expression was correlated with VISTA expression and reduced SLE susceptibility (50). These TF associations further support the concept that there are overarching tolerogenic programs induced after kidney infiltration.

Despite the clear presence of a T_EX_ population in both murine lupus models, a T_EX_ population was not observed in human LN in a report from the AMP consortium (4). Notably, due to the difficulty in obtaining tissue, the AMP study examined few T cells (just over 1000 total from all patients), and these were obtained from patients treated with immunosuppressive therapy. While it is theoretically possible that T cell exhaustion does not occur in human LN and is mouse specific (6) and human tumor specific (51), it seems more likely that human autoimmunity will resemble human tumor infiltration; thus, deeper study may reveal exhausted KITs, at least in some patients. T cell exhaustion is manifestly observed in the peripheral blood CD8^+^ cells of patients with lupus, and the presence of such exhausted cells in blood correlates with less propensity for disease progression (11, 52). If severe disease correlates with less ability to induce exhaustion, it is possible that those patients who sufficiently induce exhaustion may not be well represented in the AMP data that focused on patients who warranted (re)biopsy. Treatment with prednisone or other immunosuppressive agents may also preferentially deplete the exhausted population. Corticosteroids can induce T cell apoptosis (53) and reduce the efficacy of anti–programmed cell death 1 (anti–PD-1) therapy (47, 53, 54). Additionally, treatment with cyclophosphamide leads to a vast reduction in PD-1^+^Lag3^+^ T_EX_ in tumors and thus would likely do the same in human nephritic kidneys (55). Indeed, the efficacy of these very treatments for nephritis implies that they must be impacting KITs and the renal microenvironment.

Integrating our findings, we propose several mechanisms by which kidney damage occurs in a subacute fashion, even in the face of inherent counterregulation within the tissues. Cytotoxic like CD4^+^ T cells may contribute to damage (28, 29). Parenchymal destruction may also be caused by a combination of the early infiltrating/transitional CD8^+^ cells that recognize self-antigen and expand, before they become fully exhausted. It is further possible that the IFN^hi^ and T_EM_ cells, which are present in small numbers, are also pathogenic. Critically, while T_EX_ are dysfunctional, they are not nonfunctional (7, 51); accordingly, these cells may also contribute to the slow chronic damage we observe in LN and likely continue to respond to mitogenic stimulation, as supported by the continuing S and G2/M phase signatures seen in T_EX_ (Figure 8H).

While it does not appear that the percentage of T_EX_ increases with disease severity (Supplemental Figure 6C), or as lupus-prone animals age (6), the total number of infiltrating T cells does increase with disease severity in these models and in human patients. Thus, there may be a point beyond which exhaustion can no longer control disease. Prior work suggested that those patients with a peripheral blood CD8^+^ T_EX_ signature were less likely to flare compared with those lacking this signature (52). However, what occurs at the level of the target organ remains an outstanding question, and it will be interesting, in future work, to determine if the proportion of exhausted cells in a biopsy can be correlated with disease outcomes.

It will be important to identify the mechanisms by which each of these potentially damaging cell types and events are inhibited by the kidney and how existing and novel therapies can affect them. It is hoped that this work will allow us and others to target these varying T cell populations more specifically, thereby opening new therapeutic avenues.

## Methods

### Animals.

C57BL/6 mice were purchased from Jackson Laboratory. MRL/lpr mice were initially obtained from Jackson Laboratory and have been maintained in our laboratory. FcγR2B^–/–^.*Yaa* mice were obtained from Silvia Bolland (National Institute of Allergy and Infectious Diseases, NIH, Rockville, Maryland, USA) and have been maintained in our laboratory. Mice were aged and proteinuria was measured prior to sacrifice, as documented in Supplemental Table 2.

### Isolation of T cells from kidney and spleen.

T cells were isolated as previously described (6). In brief, after sacrifice, spleens were removed, and animals were perfused with 40 mL of HBSS, until complete blanching of liver and kidney occurred. KITs were isolated using the Octodissociator (Miltenyi Biotec) in the presence of 1600 Kunitz units/mL collagenase D (Roche Diagnostics) and 0.2 mg/mL DNAse IV (MilliporeSigma) for 30 minutes at 37°C. RBC lysis was performed, and cells were filtered through a cell strainer (100 μM nylon; Falcon, Corning). Splenocytes were isolated using mechanical dissociation between 2 frosted glass slides and filtered through a 70 μm mesh filter after RBC lysis.

Cells were stained as previously described (6) with the following panel: anti-CD8 (Lyt-2/TIB-105, Pac-Blue, produced in-house), anti-CD4 (GK-1.5, in PE, purchased from BioLegend), and anti-CD90.2 (Thy1.2/30H12, Al488, produced in-house) for MRL/lpr mice or anti-CD90.2 (Thy1.2/30H12, Al647, produced in-house) for FcγR2B^–/–^.*Yaa* Ghost BV510 (Tonbo) was used to exclude dead cells. For all “in-house” antibodies, hybridoma clones are commercially available and antibodies were purified as described (6).

T cells were sorted using a FACSAria (BD Biosciences). After sorting, cells were washed twice with 2% BSA in PBS. Then anti-CD45 HTO reagents were added at a 1:50 dilution to each of the sorted samples. Anti-CD45 TotalSeq-A0096 30-F11 (BioLegend) was used for the Main-Seq cohort, and TotalSeq-C0096 30-F11 (BioLegend) was used for both TCR-Seq cohorts. After the HTO staining cells were washed twice in 2% BSA in PBS.

### Library preparation and RNA-Seq of T cells.

Cells were counted and loaded into the 10x Genomics Chromium system per the manufacturer’s instructions. Gene expression, TCR, and antibody hashtag/feature barcode libraries were generated, their quality was assessed through the Agilent TapeStation High Sensitivity D5000 Screentape, and their amounts were quantified with the KAPA Library Quantification Kit for Illumina Platforms. For the Main-Seq cohort the 3PrimeV2 library was used; the feature barcode library was generated according to the New York Genome Center protocol (56). For the TCR-Seq cohorts the 5PrimeV1 libraries were obtained from 10x Genomics. For hashtagging, we followed the “feature barcode” instructions from the manufacturer. Libraries were pooled and sequenced on a NovaSeq (Illumina Biosciences). FASTQ files were generated and aligned to the mouse reference genome mm10 with Cell Ranger 5.0.0 (10x Genomics) to produce the gene-cell count matrix and cell-antibody count matrix.

### scRNA-Seq data processing.

The 10x raw data from each sample were demultiplexed, and FASTQ files were generated using the “mkfastq” Cell Ranger pipeline (v5.0.0, 10x Genomics). Cell Ranger “count” was used to align reads to the mm10 reference genome, and mRNA transcript, and HTO unique molecular identifier (UMI) quantification tables were generated. The raw barcode matrix files generated from the Cell Ranger pipeline were further utilized for downstream analysis using the Seurat package (v4.0.0) (12) (https://github.com/satijalab/seurat) in R (v3.4.3).

### Initial quality control and processing.

Cells expressing fewer than 200 genes, or with greater than 10% of UMIs that mapped to mitochondrial DNA, were filtered out. The HTO tables were added to the data set and normalized by a centered-log ratio method using the “NormalizeData” function. The normalized HTO count was used to determine if each gel bead-in-emulsion contained a single cell using the Seurat “MULTIseqDemux” function and manual inspection of cells, where a cell was considered a singlet if expression of a single HTO accounted for more than 70% of the total HTO expression in that cell; otherwise, the cell was considered a doublet and removed. Gene expression values for each cell were log_2_-normalized using “NormalizeData” function, where expression of a gene was normalized to total expression of all genes in that cell and scaled by a factor of 10,000. UMI, mitochondrial content, and hemoglobulin gene and ribosomal gene content scores were “regressed out” using Seurat’s “ScaleData” function.

### Dimensionality reduction and clustering.

Variable genes were detected using the “mean.var.plot” method in the “FindVariableFeatures” function with default cutoff, which computes the mean expression and dispersion (log[variance/mean]) per gene, followed by grouping the data into 20 bins based on their mean expression. Variable genes were then selected based on *z*-scored dispersion within each bin. These variable genes were used for dimensionality reduction based on principal component analysis (PCA) using the “RunPCA” function. “ElbowPlot” was used to assess the first 50 principal components, and the principal components that accounted for the largest variability in the data were selected for further UMAP dimensional reduction and clustering analysis.

In order to identify distinct groups of cells, unsupervised clustering was performed using the “FindClusters” function, which calculates the k-nearest neighbors according to variable gene expression in all cells, thereby constructing a shared nearest neighbor graph using the Louvain algorithm. To avoid overclustering, we tested different resolution (“res”) parameters, ranging from 0.1 to 2 in increments of 0.1, and the clustering progression was assessed and visualized using “Clustree” (v 0.4.3) (57). Optimal resolution was determined based on continued separation prior to “overclustering” as observed by the increasing crossover between clusters. Low-resolution clustering was then defined as analysis of all cells within an entire cohort, while high-resolution clustering was defined as clustering on a preselected subpopulation, i.e., CD4^+^, CD8^+^, or Treg cells previously defined in our low-resolution analysis. Based on these observations we chose the resolutions 0.9, 1.4, 0.6, 0.9, respectively, for Main-Seq, CD4^+^, CD4^+^ Treg sub, and CD8^+^ from cohort 1 and resolution 0.7 for merged cohort CD8^+^ T cells. Cell clusters were visualized using UMAP dimensional reduction plots.

The “FindAllMarkers” function with default settings was utilized to find DEGs in each cluster, in comparison with all other clusters, using the Wilcoxon rank-sum test with genes detected in a minimum of 10% of cells, a minimum of 0.25 average log fold change, and a minimum of 0.01 Bonferroni-adjusted *P* value.

### Harmony analysis.

For the combined UMAP in Figure 8, the 3 independently run cohorts were merged using Harmony (58) with default parameter settings.

### Cell cycle evaluation.

The “CellCycleScoring” function in Seurat was utilized to calculate the G1, G2/M, and S phase marker expression score in each cell using scoring strategy described previously (59). The number of cells determined to be in G1, G2/M, or S phase was calculated on a per-cluster basis, and deviation from overall distribution was assessed by χ^2^ analysis.

### GSEA and expression mapping.

*P* values for GSEA were determined using the Wilcoxon test for published gene set signatures as defined in Supplemental Table 1. Using “ggplot2” in Seurat, –log_10_ *P* values were plotted onto the UMAPs.

### Additional analytics.

Bar plot, dot plot, and PCA plots were constructed using ggplot2 in R. Heatmaps were generated using the “pheatmap” function in R.

### TCR data analysis.

TCR data were processed using cellranger vdj with–reference = refdata-cellranger-vdj-GRCm38-alts-ensembl-3.1.0 to assemble TCR α and β chains and determine clonotypes. Cells with 1 productive TCR (α and β) were kept for further analysis, while nonproductive TCR chains were excluded. Clonal T cells were defined as cells expressing shared TCR-α and -β receptors with identical CDR3 sequences at the nucleotide level.

### Pseudotime trajectory analysis.

Trajectory analysis using the Seurat-processed gene counts was performed using Monocle 3 (version 0.1.3) to model CD4^+^ T cell differentiation. The reverse graph embedding method DDRTree was used to reduce the dimensionality, cells were ordered along a trajectory using “orderCells” function, and the trajectory was visualized in the reduced dimensional space.

To model CD8^+^ T cell differentiation, we used Slingshot with “start.clus” set as B6/naive cluster. The cells were ordered by Slingshot pseudotime and grouped as Seurat clusters to project the clusterwise trajectory. The Slingshot trajectory lineage result was plotted on the CD8^+^ cell Seurat UMAP.

### TF regulatory network analysis.

The SCENIC v.1.1.2 workflow was utilized in R to identify regulons using the Seurat-processed count and clusters as per a previous study (60). Heatmaps were then created as described above.

### Histologic scoring.

Kidney histology preparation and scoring were performed as defined previously (61).

### Data and materials availability.

All analyses and visualizations were performed in R. Specific methodology and analysis using published Seurat programs are detailed in the Methods section. The data, metadata, and analysis outputs such as cluster identification are deposited in National Center for Biotechnology Information’s Gene Expression Omnibus (GSE197339).

### Statistics.

Statistics were calculated in R as indicated in analysis-specific sections or in GraphPad Prism by 1-way ANOVA with Tukey’s test for multiple comparisons, 2-way ANOVA with repeated measures, or χ^2^ analysis as defined in figure legends, with *P* values represented as *P < 0.05, **P < 0.01, ***P < 0.001, ****P < 0.0001. *P* < 0.05 was considered statistically significant.

### Study approval.

All work was approved by either University of Pittsburgh’s or Yale University’s Institutional Animal Care and Use Committee.

## Author contributions

JST and MJS conceived the study. SS, MC, JST, and MJS developed methodology. JST and SS investigated. JST and SS visualized data. JST and MJS acquired funding. JST and MJS provided project administration. JST and MJS provided supervision. JST and MJS wrote the original draft. JST, MJS, SS, and MC reviewed and edited the draft.

## Supplementary Material

Supplemental data

## Version 1 03/10/2022

In-Press Preview

## Version 2 04/22/2022

Electronic publication
