# Supplementary material for: Heterogeneity and clonality of kidney-infiltrating T cells in murine lupus nephritis
Source: JCI Insight. 2022 Apr 22;7(8):e156048. doi: 10.1172/jci.insight.156048 (PMC9089785; doi:10.1172/jci.insight.156048)
Supplement: Supplemental data [file jciinsight-7-156048-s110.pdf]

Supplemental Data:

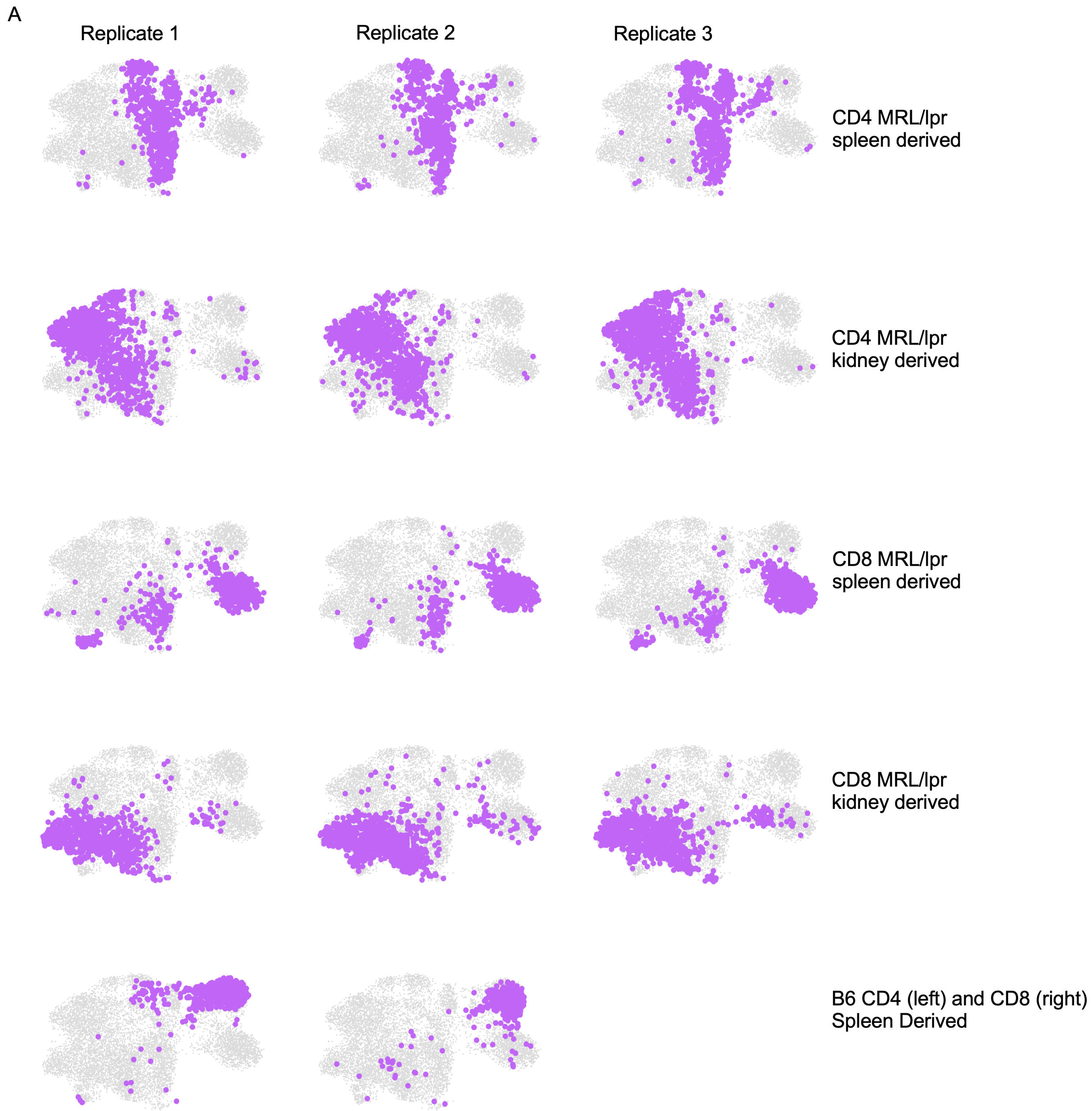

Supplemental Figure 1: *Analytics methodology* (A) Cell source determined using HTO for each of the 14 individual sorted populations was overlaid on the UMAP projection (as per Fig 1B) and organized based on replicates to exhibit the equal contribution of each replicate to the observed clusters.

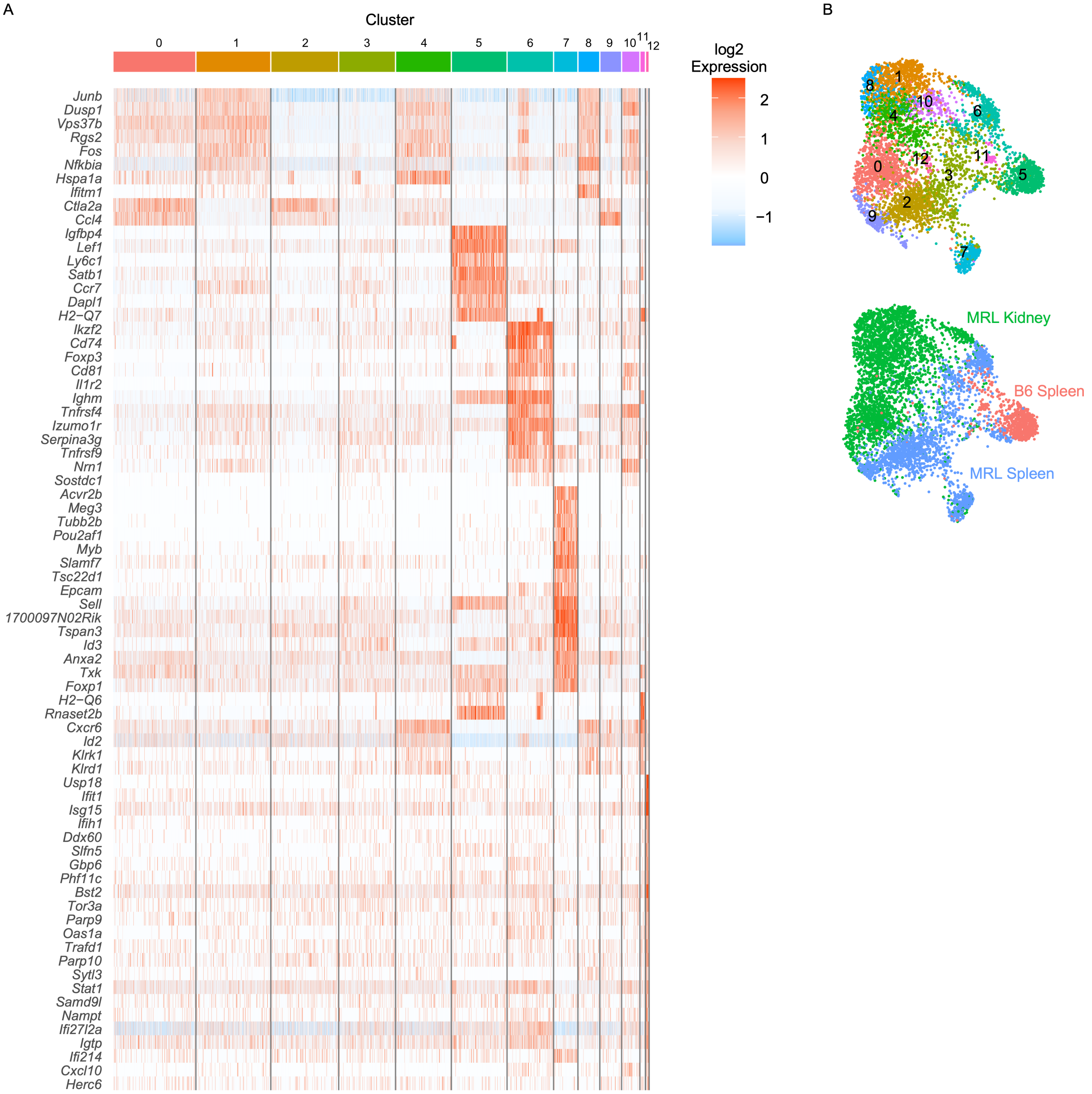

Supplemental Figure 2: *Top DEGs associated with CD4 T cell clusters.* (A) Heat map shows top (Fold change > 1 and FDR < 0.01) significant differentially expressed genes (rows) associated with the 13 CD4 clusters and their expression at the single cell level (columns), colored according to expression level. (B) With UMAP projection of CD4 T cells from Main-seq outlining 13 clusters, and overlay of cell source as determined by HTO (as defined in Figure 2).

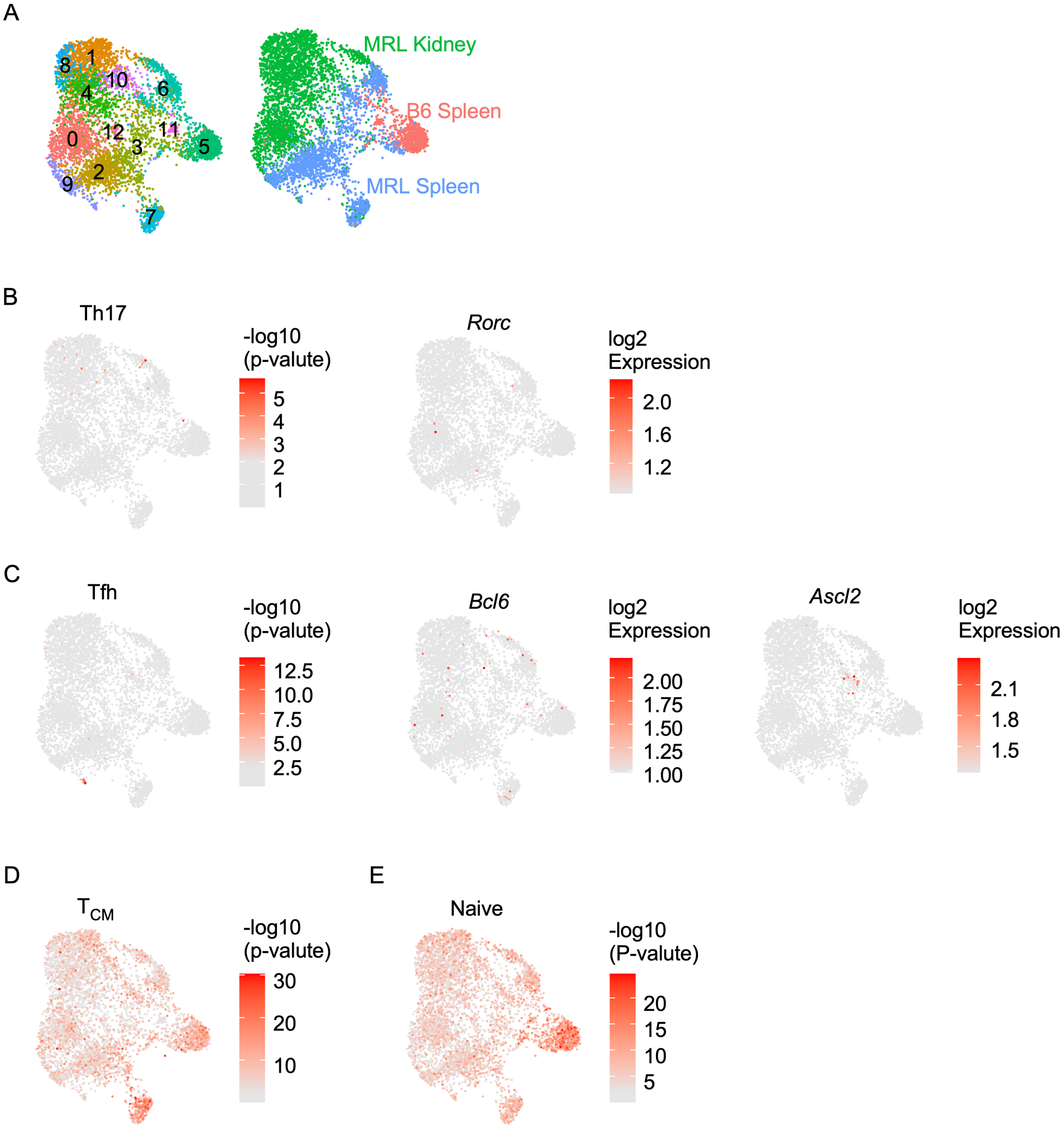

Supplemental Figure 3: *GSEA and gene expression mapping identifies clusters and cells with a naïve and T_{CM} signature but not Th17 or Tfh populations*. GSEA performed in each cell by Wilcox-test using published reference signature genes (Table S1) and related TF expression is mapped onto the (A) CD4 UMAP and HTO defined cell source to identify CD4 T cell phenotypes. (B) Th17 gene signature and associated TF *Rorc*; (C) Tfh gene signature and associated transcription factors *Bcl6* and *Ascl2*; and gene signatures for (D) T_{CM} and (E) naïve populations.

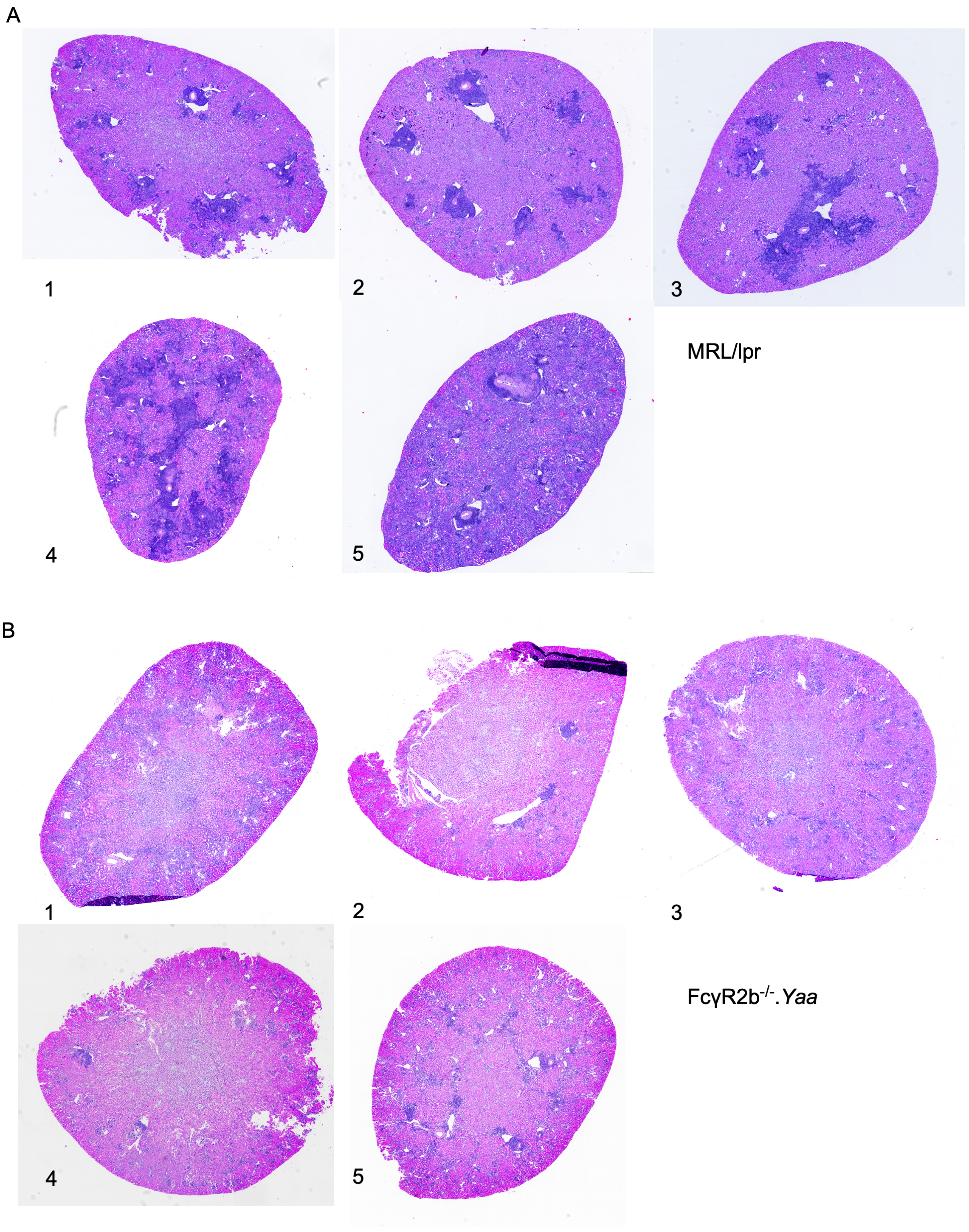
A
1
2
3
4
5
MRL/lpr
B
1
2
3
4
5
FcγR2b-/-.*Yaa*

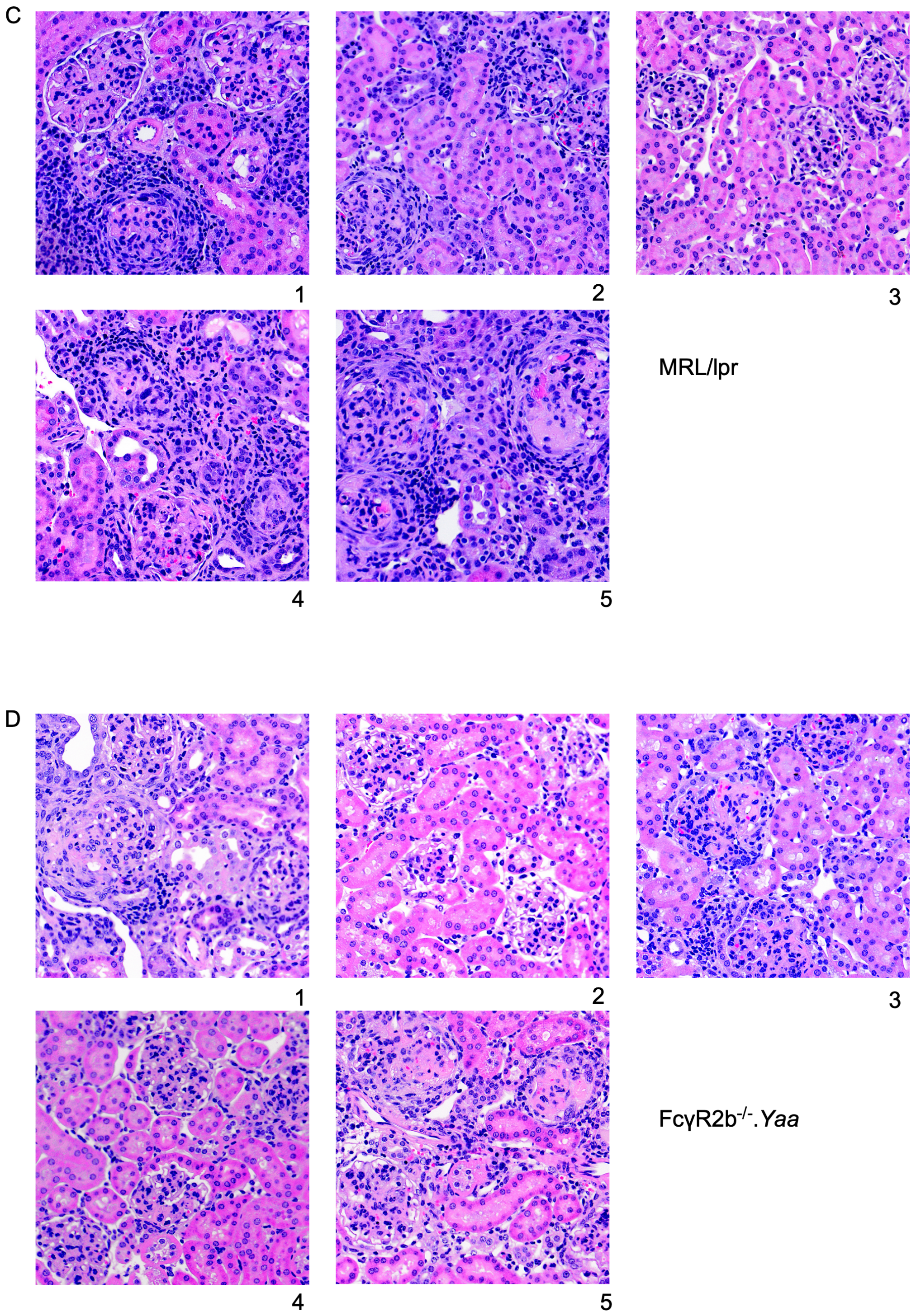

Supplemental Figure 4: H&E stained cross section of kidneys from mice analyzed in (A) cohort 2 (MRL-TCRseq), and (B) cohort 3 (Yaa-TCRseq), with high resolution 20x images of representative glomeruli for (C) cohort 2 mice (MRL-TCRseq), and (D) cohort 3 (Yaa-TCRseq). Associated disease scoring is included in Supplemental Table 2.

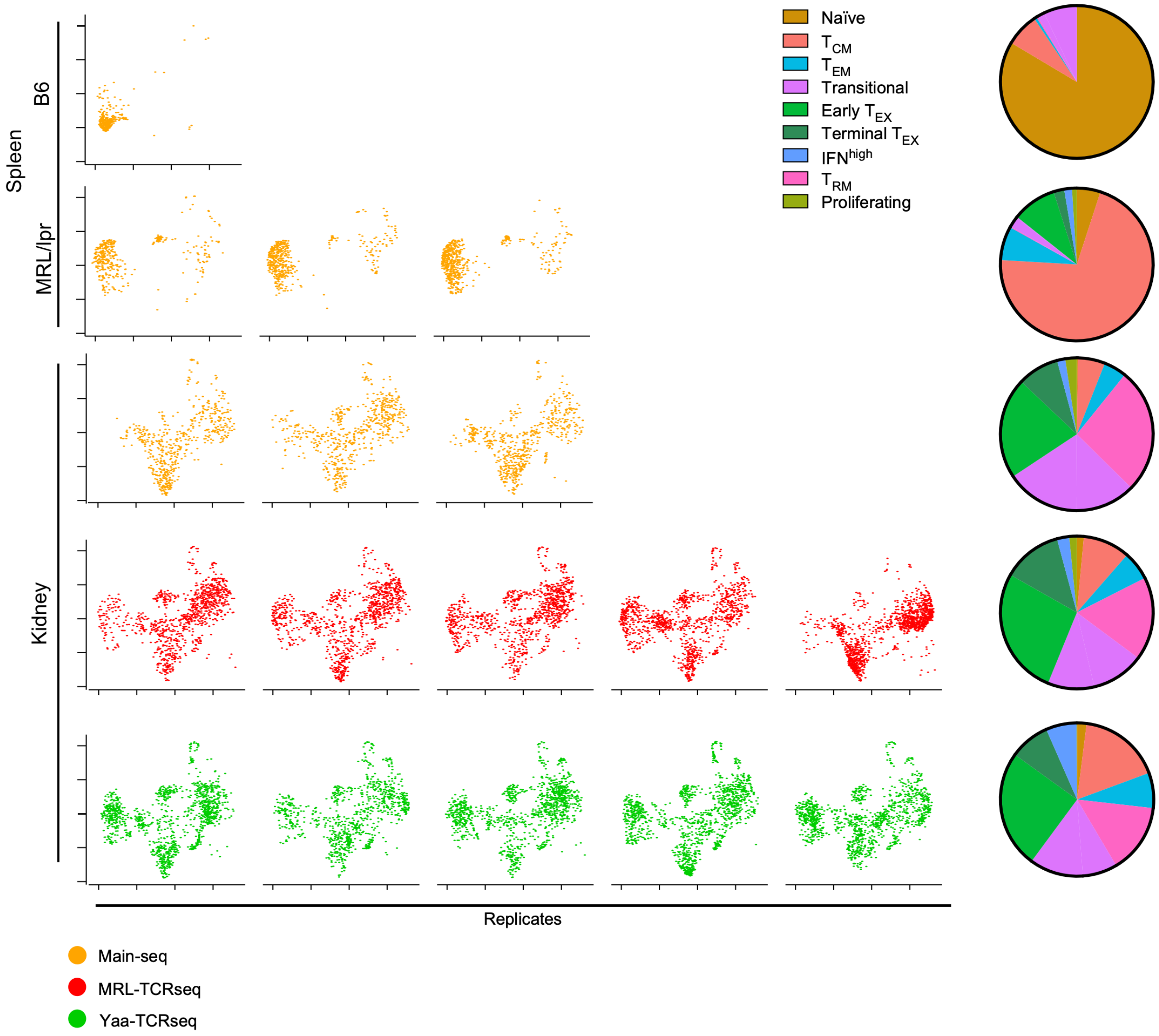

Supplemental Figure 5: *Cluster contribution by mouse and cohort.* Mouse by mouse contribution to combined CD8 UMAP cluster as defined in Figure 5. Each row of UMAP plots represents a single source and origin. The cohort derivation of each UMAP plot is color-coded according to the legend. Pie charts to the right represent relative phenotypically-identified cluster distribution of T cells within combined cells from each source/origin.

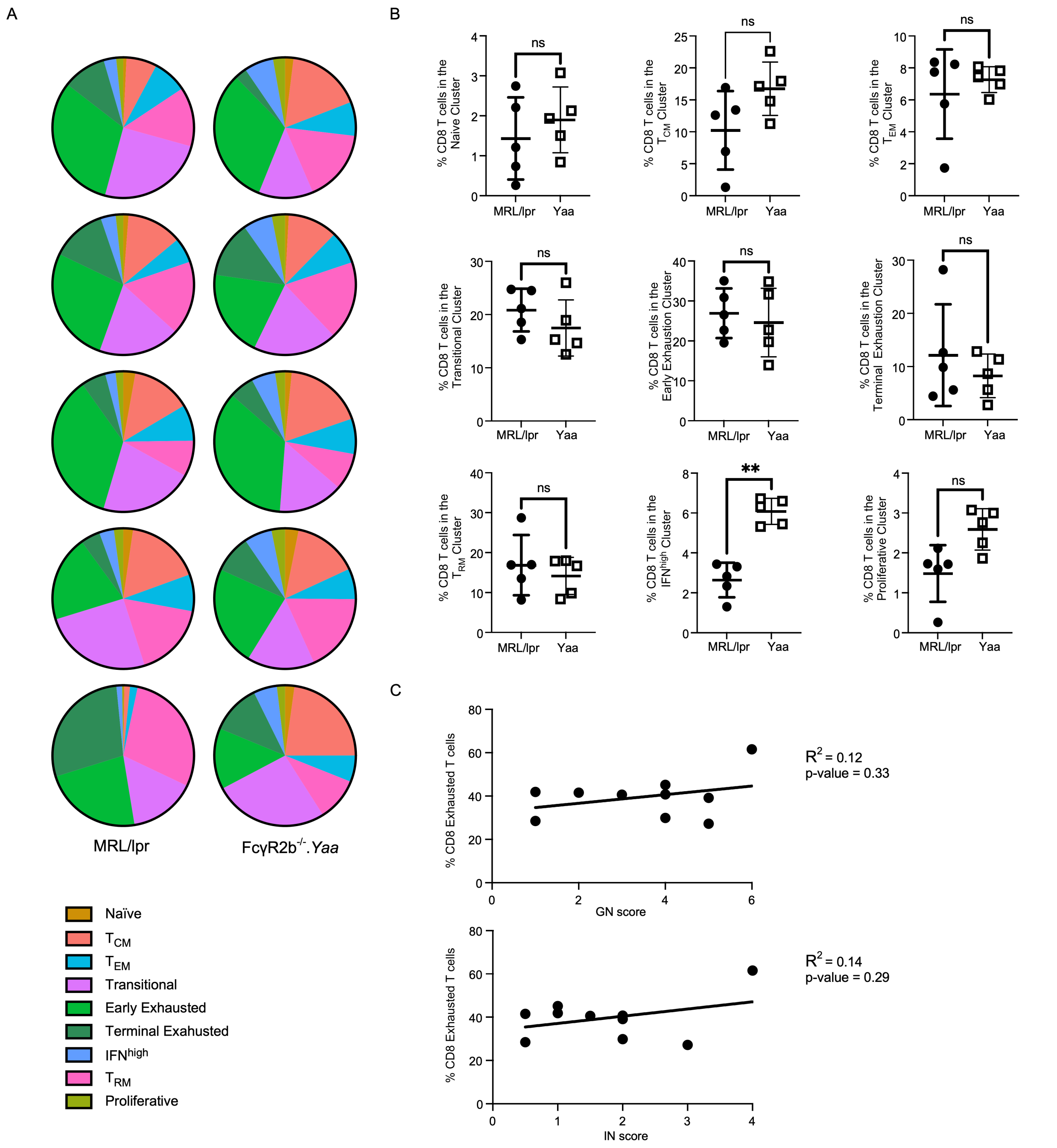

Supplemental Figure 6: *CD8 Cluster distribution does not differ by mouse model or based on histologic disease. (A)* Pie charts represent relative phenotypically-identified cluster distribution of CD8 T cells defined in Figure 5 for each mouse assessed in cohort 2 and 3. (B) Dot plot represents percent of CD8 T cells from MRL/lpr and FcγR2B$^{-/-}$.*Yaa* (Yaa) models in each outlined cluster. Statistics were determined using Two-way Anova with repeat measures corrected for multiple comparisons using Bonferroni adjustment (**

indicates p-value <0.01). (C) Linear regression analysis of % exhausted T cells in each mouse and corresponding histologic disease score, either glomerulonephritis (GN) or interstitial nephritis (IN), with no analysis reaching statistical significance.

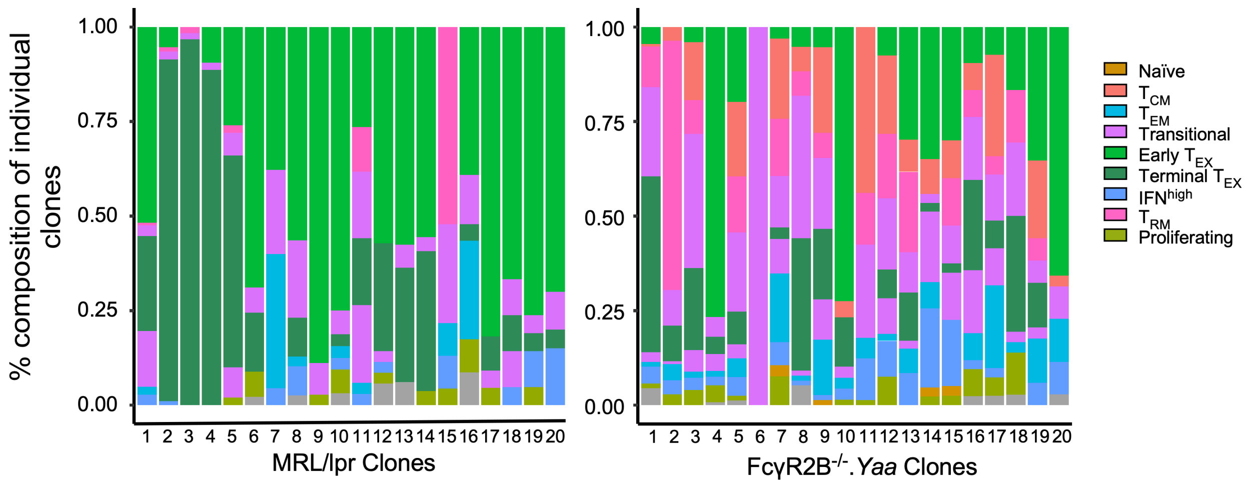

Supplemental Figure 7. *Progeny of the same T cell transition between different clusters within the kidney suggesting dynamic progression between T cell states.* Bar graphs represent the top 20 CD8 T cell clones from the MRL/lpr (left panel) and FcγR2B$^{-/-}$.*Yaa* (right panel) models with the relative distribution of these clones within the putative T cell clusters as defined in Fig. 8 B/C.

Table 1: Gene Sets used for UMAP GSEA

Profile	Cytotoxic CD4	Exhausted	Th1	Th17	Th2 long	TRM	Tperiph	Hypoxia	Treg	TEM	TCM	T naive	Tfh
**Citation**	13	14	15	15	16	17	17	18	19	20-22	20-22	21	23
**Genes**	Gzma	0610010F05RIK	Ifngr1	Ahr	Plac8	Cdh1	Sidt1	Bnip3	Foxp3	Cx3cr1	Cd28	ABLIM1	Il21
	Gzmb	0610037D15RIK	Il18r1	Irf4	Igfbp7	Cd244	Rasgrp2	Prodh	Ctla4	Id2	Tiam1	ACTN1	Plagl1
	Prf1	1110035E04RIK	Cxcr6	Nfxl1	Pdcd1	Itga1	S1pr1	Idh1	Il2ra	Stat4	Cpne2	ACVR1C	Sostdc1
	Gzmh	1190002F15RIK	Il18rap	Rora	Il5	LOC641050_	Cmah	Prodh2	Il2rb	Ccl4	Cdh1	ADD3	Ttc39c
	Nkg7	1200009F10RIK	Klrc1	Rorc	Arg1	Skil	Fam65b	Pdk2	Tnfrsf4	Ccl5	F2rl1	ADSL	Popdc2
	Gnly	1700009P17RIK	Il12rb2	Il21	Il1rl1	Sik1	Ly6c2	IDH1	Tnfrsf9	Lgals1	Sesn3	AIF1	Myo1f
	Klrg1	1700012B09RIK	Etv5	Il12	Gata3	Litaf	Elovl7	IDH2	Itgae	Lgals3	Sell	ALS2CR13	Sostdc1
	Klrb1	1700012D01RIK	Hopx	Il17f	Bhlhe40	Chn2	Eomes	BNIP3L	Ccr6	Klrk1	Ccr7	ALS2CR13	Dntt
	Itgal	1700017B05RIK	Onecut2	Il17a	Gclc	Ctla4	Fgf13	MX11	Cd83	Itgal	Cd27	AMY2B	Sccpdh
		1700019E19RIK	Tbx21	Il2	Ltb4r1	LOC641050_	Klre1	PDK1	Klrg1	Itgb1	Eomes	APBB1	Cdc25c
		1700106N22RIK	Ifng	Inhba	Rnf128	Icos	S1pr5	SLC2A1	Penk	Klrc1	Tcf7	APEX1	Rab39b
		1810009J06RIK.GM2663	Csf1	Pgf	Fgl2	Inpp4b	Slamf6	P4HA2	Cst7	Gzma	Stat3	ARH	Cxcr3
		2010107G23RIK	Il34	Il2ra	Serpinb6a	Tmem123	Tlr1	Hif1a	Ahr	Gzmb	Id3	ARMCX1	Gldc
		2010111I01RIK		Ptgfrn	Pparg	Vps37b	Usp33		Il1rl1	Prf1		ATM	Aurkb
		2210403K04RIK		Tfrc	Nfkb1	Nr4a2			Ikzf4	Il2rb		ATM	Angptl2
		2310016C08RIK		Tnfrsf13b	Ccr8	Itgae			Ikzf2	Il18rap		ATP8A2	Zdhhc2
		2310039F13RIK		Traf6	Cxcr6	Rgs1			Ebi3	Casp1		AXIN2	Pif1
		2310046K01RIK			Tagln2	Xcl1				Tbx21		B4GALT3	Aspm
		2310079F09RIK			Zcchc10	Rgs2				Prdm1		BACH2	Zdhhc2
		2500002B13RIK			Ctla2a	LOC100503878				Klf2		BEX2	Aurkb
		2610024B07RIK			Cd200r1	Nr4a1				Rora		BNIP3	Ces2d-ps
		2700007P21RIK			Litaf	Hspa1a						BTBD11	Ces2c
		2700008G24RIK			Rgs1							CABC1	Tmbim1
		2810433K01RIK			Il6							CACNA1I	Cdc25c
		2900001G08RIK			Id2							CAMK4	Ispd
		2900026A02RIK			Rgs2							CCND3	Slc29a4
		3830408D07RIK			Rgcc							CCR7	Angptl2
		4831426I19RIK			Il4							CD164L1	Slc43a1
		4930422G04RIK			Mns1							CDR2	Il1rl1
		4930429F24RIK			Msmo1							CGI-30	Perp
		4930434E21RIK			Sdf2							CHKA	Ttk
		4930520O04RIK			Paxbp1							CKAP4	Gas2l3
		5330409N07RIK			Gm5127							CLNS1A	6330403K07Rik
		5330426P16RIK			Il17rb							CNN3	Ifng
		5730469M10RIK			Hlf							COH1	Melk
		5830415L20RIK			Fam213a							CPT1B	Coro2b
		5830456J23RIK			Il13							CRLF3	Aspm
		5830474E16RIK			Dusp6							CTL2	Chst2
		6030487A22RIK			Lgals3							CUGBP2	Cenpe
		6230409E13RIK			Epas1							DAF	Osbpl3
		6430537I21RIK			Tnfsf11							DEAF1	Zdhhc2
		9230110C19RIK			Plk3							DGKA	Il2
		9230116N13RIK			Stx12							DHRS3	Klrc1
		9430037O13RIK			Cysltr1							DKFZP434B	D930003E18Rik
		9430087J23RIK			Egr1							DKFZP564M	LOC102641248
		9530053H05RIK			Tusc3							DOCK10	Maf
		9630010G10RIK			Rbpj							DSCR1L2	Alcam
		9630013D21RIK			Aprt							EFHC1	2810417H13Rik
		A930012M21RIK			Ldha							EIF2S3	Cadm1
		A930012O16RIK			Samsn1							EIF3S6	Cebpa
		A930026I22RIK			4932438H23Rik							ESD	S100a8
		AA407881			Degs1							EZH1	Alcam
		AA408396			Adck5							FCER1G	Tpx2
		ABCB1B			Ramp1							FKBP5	LOC102641248
		ABCB9			Cib2							FLJ00133	Maf
		ACADSB										FLT3LG	Dclk1
		ACTR3B										FOXP1	Angptl2
		ADAMTS6										FRAG1	Acvrl1
		ADSSL1										FXYD5	Rad54b
		AGL										FYB	Nusap1
		AI120166										GCN5L2	Cd109
		AI836003										GPR155	Clec4b1
		AI845619										GPRASP1	Tbx21
		AIFM2										HA-1	Rab26
		AK3L1.LOC100047616										HABP4	Il18rap
		AKR1B8										HAPLN3	Lrr1
		AKTIP										hIAN2	Srpk3
		ALG6										hIAN6	Tpx2
		ALS2CR4										HIPK1	Rora
		AMIGO1										HIST1H4E	Enpp2
		AMOTL2										HNRPDL	Lancl3
		ANGPTL2										HOOK1	Ryk
		ANKRD46										HPCAL4	Synpo
		ANUBL1										HRMT1L1	Mpp2
		ANXA5										HSF2	Igfbp4
		APBB1										HSPBAP1	Npdc1
		APLP1										ICAM2	Cenpf
		APOLD1										IL23A	Myo1f
		AQP1										IL6ST	Dhrs3
		AREG										IMAP1	Gm9861
		ARFGAP3.LOC100045474										IMPDH2	Litaf
		ARG1										INADL	Cxcr5
		ARHGAP21										INPP5A	Gm5593
		ARHGEF5										IRS2	Ccnb1
		ARHGEF9										ITGA6	Cela1
		ARL4D										ITPKB	Txlnb
		ARNT2										JAK3	Kdelc2
		ARPC1B.GM5637										KCTD12	Dclk1
		ATF4										KIAA0303	5330417C22Rik
		ATF5										KIAA0582	Gpr83
		ATG16L2										KIAA0748	Gng3
		ATHL1										KIAA0922	Aicda

ATL3
ATP10A
ATP2B2
AV249152
AW555355
B230312A22RIK
B3GALT5
B4GALT1
B4GALT5
B830008J18RIK
B930095G15RIK
BACE2
BAIAP2L1
BARD1
BB557941
BC010981
BC023744
BC046404
BC068157
BCL2L1
BCL2L15
BCL6B
BCMO1
BEND6
BMPR2
BNIP3
BRCA2
BSPRY
BTBD10.LOC100047893
C130057M05RIK
C1QL3
C230098O21RIK
C76533
C77545
C80258
CABLES1
CALCB
CALD1
CAMK2N1
CAMK4
CAPSL
CAR13
CAR6
CAV1
CAV2
CCDC112
CCDC122
CCDC21
CCDC34
CCL1
CCL27A
CCR8
CD200R4
CD34
CD70
CD93
CDC14A
CDC14A.LOC100047731
CDC25C
CDK6
CENPA
CENPI
CENPP
CETN4
CHAC1
CHST10
CHST12
CIAPIN1
CILP2
CKAP2
CLDN12
CLEC4D
CNNM2
CNRIP1
CNTLN
COCH
COL1A2
COPZ2
CPA2
CPE.LOC100046434
CRYBG3
CSF2
CTNND2.LOC100045979
CTSC
CTSD
CXCL1
CXCL2
CXCL3
CXCR6
CYR61
D17ERTD165E
D18ERTD289E
D330040H18RIK
D3ERTD751E.GM9529
D430033H22RIK
D5WSU178E

KIAA1078	Mki67
KIAA1189	Ndrg4
KIAA1729	Kif4
KIAA1971	Osbpl3
KIF2	Ccnb2
KIFAP3	Endod1
KLF7	Birc5
LASS6	Endod1
LCHN	Cnn3
LDHB	Fads2
LEF1	Srxn1
LENG10	Kif11
LEPROTL1	S100a4
LETMD1	Zbtb32
LGTN	Lacc1
LIAS	Pbk
LISCH7	8-Mar
LKAP	Tnfsf14
LNPEP	Gm3924
LOC129293	Endod1
LOC143903	Bmp7
LPAAT-e	Gm34221
LRRC5	Ctla2b
LRRN3	Alcam
LTA4H	Myo1f
LTB	Plek
LY96	Optn
LYPLAL1	Srxn1
MAGEH1	Gstt3
MAL	Gm5593
MAML2	Ccnb1
MAN1C1	Zdhhc2
MDS2	Bub1
MEST	Jdp2
MGC15763	Tmem2
MGC17330	8030451A03Rik
MMS19L	Chrdl1
MPHOSPH9	Cdca8
MRPL1	Soat2
MSL3L1	S100a6
MTRF1	Atp1b1
MTUS1	Morn4
MYB	Klrc1
MYC	Spag5
NDE1	Tjp2
NDFIP1	Igfbp4
NDUFB5	Ccna2
NELL2	Ampd1
NGFRAP1	Runx2
NKX3-1	Stfa3
NOG	Igfbp4
NRCAM	Gm17619
NUCB2	Tshz2
PCSK5	Gpm6a
PDK1	Ncapg
PHEMX	Tk1
PIK3CD	Kitl
PLAC8	Morc1
RAC2	Dtl
RAPGEF6	Aifm2
RASA2	Gm36401
RASA4	Adam19
RIN3	Stil
RNU22	Kif14
SAMHD1	Igfbp4
SP110	Ccnb1
STK4	Gzmk
STX16	Proser2
SUMF2	Ccna2
T3JAM	Rragd
TM4SF8	Pawr
TPP2	Pif1
TTC11	Ccnf
USP52	Esco2
VPS35	2810417H13Rik
WDR42A	Gna15
ZFP90	
ZNF291	
ZNF44	

D9ERTD26E
DAB2IP
DCLK1
DCN
DEGS1
DENND4A
DEPDC1A
DLG2
DMXL2
DNAJC12
DNAJC25
DPYSL3
DRAM1
DSC2
DSCAM
DUSP16
DXERTD242E
E2F7
E330009J07RIK
EIF2AK2
ELK3
ELOVL4
ENO2
EPAS1
EPS8L3
EREG
EXPH5
F13A1
F630043A04RIK
FAM110A
FAM178B
FAM19A3
FAM20C
FAM54A
FAM5C
FAM64A
FANCC
FANCD2
FANCF
FARP1
FARS2
FAT1
FBXO11
FCHO2
FGFBP3
FHL2
FILIP1
FKBP7
FLOT2
FN1
FOXF1A
FOXG1
FOXM1
FOXRED2
FRMD4A
FRZB
FST
FTSJD1
FUCA2
FZD4
FZD6
GABRR1
GALC
GALNT3
GALNTL4
GAN
GAS2
GGTA1
GIPC2
GJA1
GJB2
GLDC
GLP1R
GLT8D3
GM14057.PPP1R14C
GM14288
GM5188
GM6194.TMEM189
GM9861.LITAF
GNB5
GOLGA7
GPD2
GPLD1
GPR160
GPR177
GPR65
GPR97
GRB10
GREM1
GRP
GSTM5
GSTT1
GSTT3
GTF2IRD1
GZMD
GZMD.GZME

GZME
GZMF
GZMG
H2.Q5
HBEGF
HCFC2
HECTD2
HHAT
HIGD1A
HIP1R
HISPPD1
HIVEP1
HMGA2
HMMR
ICA1
IDI2
IFI204
IGF2R
IGFBP7
IGSF5.PCP4
IL1R2
IL1RL1
IL33
IMPA2
IQGAP3
IRAK1BP1
IRG1
ITGAV
ITIH5
ITLN1
JAM2
JAZF1
KAZALD1
KCNA4
KCNF1
KCNK10
KCTD9
KIF13B
KIF18A
KIF24
KIF4
KIFC1.LOC100044746
KIFC3
KLRA7
KLRB1A
KLRC2
KLRD1
KLRE1
KRT20
LAMB1.1
LANCL1
LGALS7
LOC100044896.SCCPDH
LOC100046560.MAGED2
LOC100047565.MMD
LOC633966
LOC640441.THBS1
LOC641050
LOC677224
LPAR1
LTBP1
LTBP3
LTF
LXN
MAGOHB
MAPK6

MARVELD2
MASTL
MED12L
MELA
MGP
MLF1
MLKL
MMD
MOBKL1A
MORN3
MORN4
MRC2
MT2
MTAP2
MTAP6
MTHFS
MTMR7
MUC1
MUC20
MXD3
MXI1
MYLK
MYO10
MYO16
MYO1E
NAP1L3
NCAM1
NCOR2
NDFIP2

NEDD9
NEIL3
NELF
NFAT5
NFATC1
NHEDC2
NID1
NMT2
NPHS2
NPNT
NR3C1
NSBP1
NSMAF
NT5E
NUDT11
NUPR1
OBFC2A
OCIAD2
ODZ4
OMD
OPTN
ORC1L
OSGIN1
OSR2
OSTF1
OTX1
OVOL2
PADI2
PAQR3
PASK
PAWR
PBX3
PCK2
PCOLCE
PCYT1A
PDCD1
PDGFB
PDPN
PEAR1
PENK
PERP
PGGT1B
PHACTR3
PI4K2B
PIWIL2
PKD2
PLA1A
PLAGL1
PLD2
PLOD2
PLSCR4
PON3
PPP1R16B
PPP1R3B
PRF1
PRICKLE2
PRKG2
PRR15
PRRX1
PTER
PTGS1
PTPLAD1
PTPLB
PTPN3
PTPRK
RAB19
RAB39B
RABGEF1
RAD54B
RAI14
RALGDS
RAPH1
RAPSN
RASD2
RBP1
RBPJ
REM2
RETSAT
RGS8
RHOQ
RHOU
RHOX5
RNF128
RPA2
RPGR
RYK
SAG
SDCBP2
SDF4
SDPR
SECTM1A
SEMA4C
SEMA6D
SEPHS1
SERPINB6A
SERPINB9B

SGMS2
SGOL2
SIGLEC5
SLC15A1
SLC16A11
SLC16A3
SLC16A4
SLC17A6
SLC22A15
SLC25A19
SLC2A8
SLC30A9
SLC37A2
SLC39A14
SLC5A3
SLC6A12
SLC7A3
SLFN3.SLFN4
SMPD4
SMPDL3B
SMYD1
SNX16
SOX9
SPA17
SPAG5
SPAG9
SPARC
SPATS2
SPDYA
SPIN4
SPIRE1
SPO11
SPP1
SRXN1
SSBP3
ST14
ST6GALNAC3
ST6GALNAC6
STARD4
STFA3
STK38L
STK40
STYK1
SULT2B1
SUOX
SUV39H2
SYNC
SYTL3
TEAD1
TEX15
TFF1
TG
THBS1
THBS2
TIMP1
TJP1
TJP2
TM4SF1
TMED8
TMEM120B
TMEM135
TMEM158
TMEM159
TMEM170B
TMEM171
TMEM184C
TMEM218
TMEM30A
TMPRSS6
TNFRSF18
TNFSF13B
TNFSF4
TPBG
TPK1
TPMT
TRAIP
TRIM36
TRIM72
TRIP10
TROVE2
TRPC1
TTK
TULP4
TUSC3
TWIST1
TWSG1
UBASH3B
UHRF2
ULBP1
UPP1
VASH1
VAT1L
VLDLR
VPS54
WISP1
WWC1

WWTR1
ZBTB25
ZCCHC14
ZFP580
ZFP862
0610010B08RIK.GM14295.LOC627901
1110004F10RIK
1110067D22RIK
1300014I06RIK
1500009L16RIK
1700025K23RIK
2010002N04RIK
2410127L17RIK.LOC677553
2510009E07RIK
2600001M11RIK
2610027H17RIK
2610029I01RIK
2610318N02RIK
2700097O09RIK
2810417H13RIK
3110073H01RIK
4921509J17RIK
4930547N16RIK
4930551O13RIK
4933413G19RIK.FOXM1.PEBP1
6330503K22RIK
A430093F15RIK
A630035D09RIK
AA467197
ABHD14A
ABHD4
ABI2
ACADL
ACOT7
ADAM9
ADM
ADRB1
AI747699
AI847670
ALAD.LOC100046072
ALCAM
ALDOA
ALDOC
ANXA1
ANXA2
ANXA3
ANXA4
AP1S2
ARF2
ARHGAP18
ARL3
ARL6
ARPC1A
ARSB
ASNS
ASPM
ATAD5
ATP6V0E2
ATRNL1
ATXN1
AW555464
AXL
B3GALTL
BCAT1
BCL2A1A.BCL2A1B.BCL2A1D
BHLHE40
BIRC5
BST1
BUB1
BUB1B
C030046G05
C1QTNF6
C330027C09RIK
CALM3
CALU
CAPG
CAR5B
CARHSP1
CASP1
CASP3
CASP4
CCDC109B
CCDC50
CCL3
CCL4
CCL5
CCNB1
CCNB1.GM5593.GM8416
CCNB2
CCR5
CCRL2
CD14
CD160
CD200
CD244
CD244.LOC677008

CD38
CD81
CDC27
CDC2A
CDCA2
CDCA3
CDCA5
CDCA8
CDKN1A
CDKN2B
CDKN3
CENPF
CENPH
CENPN
CEP55
CHN2
CHSY1.LOC100047167
CISD3
CLIP3
CMTM7
CNIH
COBLL1
CREB3L2
CRYZ
CSDA
CSF1
CSRNP1
CST7
CTLA2A
CTLA2A.CTLA2B
CTLA4
CUGBP2
CWC15
CX3CR1
CYP20A1
CYP4V3
CYSLTR2
CYTSB
D1ERTD83E
D2ERTD750E
D630039A03RIK
DAPK2
DCTN4
DCXR
DDX28
DENND3
DIAP3
DKKL1
DPY19L4
DTL
DUSP14
DUSP3
DUSP4
DUSP6
DUT
DYNLT3
E2F3
E2F8
ECE1
ECT2
EFHD2
EGR1
EHBP1
EHD4
EMILIN2
ENDOD1
ENO1.GM4735.GM5506.LOC100047043
ENTPD1
EPDR1
ERGIC1
ERMP1
ERN1
ERO1L
ESCO2
ETV5
EXO1
FAH
FAM124B
FAM162A
FAM176B
FAM72A
FAM83D
FAM92A
FAM98B
FASL
FBXO45
FCER1G
FGL2
FLYWCH1
FOS
FOSL2
FZD5
GABARAPL1
GAPDH.GM10284.GM10358.GM12969.GM2076.GM2308.GM2451.GM2606.GM3222.GM5674.GM6283.GM6322.GM8349.LOC100044981.LOC100045908.LOC100047352.LOC100048329
GATA3
GBE1

GCNT1
GCSH
GDAP2
GEM
GEMIN8
GINS1
GLRX
GM10397
GM10786
GM12397.KDM2B
GM9790.HIGD1A.LOC100045763
GMEB2
GPM6B
GPR174
GPT2
GRK5
GSTO1
GZMC
HIF1A
HIP1
HIST1H2AD
HIST1H2BC
HSPA13
HSPA2
HTATIP2
ICOS
ID2
IER3
IFITM2
IFITM3
IFNG
IKZF2
IL18RAP
IL2RA
INO80C
IPO8
IRF4
ITGA1
JDP2
KCNQ5
KCTD17
KDELC2
KDM2B
KDM4D
KIF20A
KIF22
KIF2C.LOC631653
KIT
KLF10
KLRA3.KLRA9
KPNA2
KRT18
LAG3
LAMC1
LANCL3
LASS6
LAT2
LCLAT1
LCMT1
LGALS3
LITAF
LOC100046168.NDFIP1
LOC100046232.NFIL3
LOC100046643.SPRY1
LOC100047091.TMEM163
LOC676654.LYN
LOC677224.UBASH3B
LOH12CR1
LPGAT1
LPXN
LRRK1
LYZ1
LYZ2
MDFIC
MEST
METTL7A1
MID1IP1
MID2
MND1
MRC1
MRPS6
MS4A6D
MT1
MTHFD2
MXRA7
MYADM
MYST4
NAIP2
NCAPG2
NCAPH
NDC80
NDRG1
NEBL
NEK2
NEK7
NIPA2

NKG7
NLRX1
NOL7
NQO2
NR4A2
NRGN
NRN1
NRP1
NT5DC2
NUF2
OLFM1
OSBPL3
PAQR4
PDCD1LG2
PF4
PGK1
PGLYRP1
PGM2
PHACTR2
PHACTR4
PHLPP1
PIK3CG
PKM2
PLEK
PLEKHB2
PLEKHF1
PLEKHO2
PLK1
PLS3
PLSCR1
PLXDC2
PMAIP1
PPAP2C
PPFIBP1
PPP3CB
PRC1
PRDM1
PROS1
PRR11
PRSS2
PTGER2
PTGFRN
PTGR1
PTGS2
PTPN11
PTPN13
PTTG1
PXMP2
PYGL
RAB31
RABL5
RAD51
RAP2A
RASGEF1B
RFC3
RGS16
RHOC
RNF216
RRAGC
S100A1
S100A11
S100A4
S100A8
S100A9
SAMSN1
SAT1
SCD2
SDC1
SDC4
SEC23A
SELM
SEPN1
SERPINA3G
SERPINB1A
SERPINB6B
SERPINB9
SERPINE2
SESTD1
SH2D2A
SH3BGRL
SH3RF1
SHCBP1
SLC15A3
SLC25A13
SLC25A24
SLC2A3
SLC35B1
SLC35F5
SLC43A3
SLCO4A1
SLFN3
SMC2
SNRNP25
SNX9
SPOCK2
SPRY2

SRGAP3
STARD3NL
STIL
STK24
STK39
STX11
SYNPO
SYTL2
TACC3
TBC1D8B
TBX21
TCRG.V2.TCRG.V3
TCRG.V4
TCTEX1D2
TFG
TIAM1
TIRAP
TK1
TMBIM1
TMCC3
TMEM126A
TMEM38B
TMEM48
TMEM49
TMSB15B1.TMSB15B2.TMSB15B2
TNF
TNFRSF4
TNFRSF9
TNFSF10
TNFSF11
TNFSF9
TOX
TPI1
TPX2
TRAM1
TRIB3
TRPS1
TSGA10
TTC39C
TUBB6
TYMS.TYMS.PS
UBE2C
UCHL5
UGP2
UGT1A1.UGT1A10.UGT1A2.UGT1A5.UGT1A6A.UGT1A6B.UGT1A7C.UGT1A9
UNC119
UNC119B
UTF1
VAMP8
WBP5
XCL1
XDH
ZBTB32
ZC3H12A
ZC3H6
ZCCHC24
ZDHHC2
ZDHHC5
ZEB2
ZFP316
ZFP511
ZFP52
ZWILCH
1110007C09RIK
1810011H11RIK
2300004M11RIK
2310031A07RIK
2900057E15RIK
3110021A11RIK
4933424G06RIK
6430531B16RIK
A930038C07RIK
ACAD11.NPHP3
ADAM8
AF251705
AF529169
AGTRAP
AIF1
ALOX5
ALOX8
ANKRA2
ANKRD39
ANPEP
APOC2
APOE
APP
ARG2
ARMC1
ARMC3
ARSK
ATCAY
B4GALNT2
B4GALT7
BASP1.LOC100045716
BC004728
BIRC1F

C1QA
C1QC
C230073G13RIK
C3AR1
C4B
C5AR1
C79607
C920021A13
CALCA
CBARA1
CCL12
CCL2
CCL6
CCL7
CCL8
CCL9
CCNB1.GM4870.GM5593.GM8416.LOC635091
CCND1
CCR1
CD200R1
CD40
CDH17
CEP170
CERCAM
CKB
CLDN14
CLEC4A2
CLEC4A2.CLEC4B1
CLEC4A3
CLEC4E
CLEC5A
CLEC7A
CLIP2
CMA1
CMTM3.LOC100046883
COL4A4
COMMD5
CPA3
CRAT
CSF2RA
CTTNBP2NL
CXCL11
CXCL16
CXCL9
CYP4F16
D030028M11RIK
D430041D05RIK
DAB2
DDEF2
DIO2
DLL1
DUSP9
EIF2AK1
ELAC1
EPS8
FAM149A
FAM69B
FBXO31
FCGR1
FCGR2B
FCGR3
FCGR4
FOXD2
FPR2
FTL1.FTL2.GM10116.GM12164.GM5213.GM5278.LOC434624.LOC630762
GAB2
GABBR1
GATM
GDA
GDPD5
GFRA2
GM11428
GM5113
GPR56
GPR84
GSG2
GTPBP3
HAVCR2
HTR7
IGF2BP2
IGH.IGH.2.IGH.VJ558.LOC544903
IGSF6
IL1B
IL1RN
ITGA5
KIF20B
KLHL30
KNDC1
LDLR
LGMN
LILRB3
LIPE
LMBRD1
LOC100038947.SIRPB1
LOC100045163.PLBD1
LOC100048154.OTOA

LRP1
MAFB
MCPT4
MCPT8
MGAT3
MLST8
MMP12
MMP14
MS4A6C
MS4A7
MSR1
MSRB3
MTBP
NAIP5
NBEAL2
NCSTN
NDUFB4
NEK8
NHEJ1
NPL
NUDT10
OIT3
OSM
P2RY6
PCGF2
PDDC1
PDLIM7
PDXDC1
PEX11B
PI4K2A
PILRA
PLAU
PLCD1
PLEKHF2
PLEKHO1
PLXNB2
PLXND1
PPAP2A
PPP1R14A
PPP2R2C
PRR5L
PTRF
PXN
QSOX1
RAB11FIP5
REEP4
RENBP
RHBDF2
RIBC1
RIPPLY3

SFPI1
SH2D5
SLC11A1
SLC13A3
SLC16A13
SLC6A9
SLC7A8
SPATA2L
SRCRB4D
ST3GAL5
STAB1
TBXAS1
TGFBI
TIFAB
TLR2
TLR4
TMEM106A
TMEM119
TMEM208
TPSAB1
TPSB2
TREM2
TRIM45
TSPAN33
TSPAN6
TTLL10
USH1C
VPS18
WDR60
WDR67
ZFHX3
ZFP704
ZFP710
ZFP90
ZNHIT2
1110032A03RIK.LOC100048251
1700019D03RIK
1700029I15RIK
2310001H17RIK
2810039B14RIK
3110082I17RIK
4833420D23RIK
4930595D18RIK
4932415G12RIK
5330433J24RIK

5430439M09RIK
9130009I01RIK
9330180L21RIK.SFMBT1
A130009E19RIK
A230083N12RIK
AA407331
ACSL5
ACTR10
AI450241
AIM2
ALG8
ANKLE1
APOBEC3
AQP11
ARMC7
AW552393
B230354K17RIK
BBS12
C430042M11RIK
C530028O21RIK
C78115
CBR4.LOC100046395
CCDC14
CCDC58
CCDC99
CCHCR1
CD22
CD79A.LOC100047815
CD80
CLSTN1
COMT1
COX17
CRABP2
CRTAM
CSTAD
D10627
D10WSU159E
DCI
DIP2A.LOC100044059
DNAJC24
DOCK7
E030047P09RIK
E130112N10RIK
EBAG9
EEA1
EFCAB7
EHD2
ELMO2
ENO3
ENPP2
EPHB6
FAM129B
FAM188A
FAM57A
FHL3
FKBP1A
FUT7
GAS2L1
GCNT7
GH
GM11110
GM885
GNG11
GNG3
HBA.A1.HBA.A2
HIGD1C.METTL7A1.METTL7A2
HIGD1C.METTL7A2
HNRPLL
HSPB9
IDE
IDH3A
IL15RA
IL2
INTS9
IPP
ITGAL
KCND3
KCTD11
LOC100045146.RANBP9
LOC100047840.STAP1
LOC100048604.NINJ2
LOC73899
LPAR3
LPCAT4
LRRC20
LRRC4
LYRM1
MMGT2
MOSPD2
MPZL2
MRGPRE
MRPS36
MSC
MTMR1
NDUFAF2
NDUFC1

NFKBIL1
NME7
NSL1
OAZ2
ORC4L
PEX11A
PHF23
PHKA2
PIGN
PLEKHH1
POLH
POLK
POPDC2
PRKACA
PRMT1
RAB27A
RAB7
RIN3
RNASEH2C
RNF170
RPS6KC1
S100A10
SDCCAG10
SRP54A.SRP54B.SRP54C
SUDS3
SYT11
TARSL2
TBC1D2B
TBC1D7
TM9SF3
TMBIM4
TMEM180
TMEM29
TMEM39A
TNFSF14
TRAPPC1
TRAPPC4
TRPT1
TSEN2
TSGA14

Supplemental Table 2:

Experimental Mice used

Main-seq

Background	Mouse number	Age at Sacrifice	Proteinuria	Gender
MRL/lpr	1	21 weeks	4	F
MRL/lpr	2	21 weeks	2.5	F
MRL/lpr	3	21 weeks	1	F
C57Bl/6	4	8 weeks	0	F

MRL-TCRseq

Background	Mouse number	Age at Sacrifice	Proteinuria	Gender	GN score	IN Score
MRL/lpr	1	20.5 weeks	4	F	3.5	1.5
MRL/lpr	2	21 weeks	2.5	F	3	1.5
MRL/lpr	3	21 weeks	1	F	2	1
MRL/lpr	4	25.5 weeks	4	F	5	2.5
MRL/lpr	5	25.5 weeks	4	F	6	3

Yaa-TCRseq

Background	Mouse number	Age at Sacrifice	Proteinuria	Gender	GN score	IN Score
FcγR2b$^{-/-}$.*Yaa*	1	23.5 weeks	4	M	5	2
FcγR2b$^{-/-}$.*Yaa*	2	24 weeks	4	M	2	0.5
FcγR2b$^{-/-}$.*Yaa*	3	23.5 weeks	2.5	M	4	1
FcγR2b$^{-/-}$.*Yaa*	4	23.5 weeks	3	M	3	0.5
FcγR2b$^{-/-}$.*Yaa*	5	23 weeks	2	M	4	2
